# Expression QTL mapping in regulatory and helper T cells from the BXD family of strains reveals novel cell-specific genes, gene-gene interactions and candidate genes for auto-immune disease

**DOI:** 10.1186/1471-2164-12-610

**Published:** 2011-12-19

**Authors:** Rudi Alberts, Hairong Chen, Claudia Pommerenke, August B Smit, Sabine Spijker, Robert W Williams, Robert Geffers, Dunja Bruder, Klaus Schughart

**Affiliations:** 1 Department of Infection Genetics, Helmholtz Centre for Infection Research & University of Veterinary Medicine Hannover, Inhoffenstr. 7, D-38124 Braunschweig, Germany; 2 Research Group Immune Regulation, Helmholtz Centre for Infection Research, Inhoffenstr. 7, D-38124 Braunschweig, Germany and Research Group Infection Immunology, Department of Medical Microbiology, Otto-von-Guericke-University Magdeburg, Leipziger Straße 44, D-39120 Magdeburg, Germany; 3 Department of Molecular and Cellular Neurobiology, Center for Neuroscience and Cognitive Research, Neuroscience Campus Amsterdam, VU University, De Boelelaan 1085, 1081 HV Amsterdam, The Netherlands; 4 Department of Anatomy and Neurobiology, University of Tennessee Health Science Center, Memphis, Tennessee, USA; 5 Research Group Genome Analytics, Helmholtz Centre for Infection Research, 38124 Braunschweig, Germany

## Abstract

**Background:**

Regulatory T cells (Tregs) play an essential role in the control of the immune response. Treg cells represent important targets for therapeutic interventions of the immune system. Therefore, it will be very important to understand in more detail which genes are specifically activated in Treg cells versus T helper (Th) cells, and which gene regulatory circuits may be involved in specifying and maintaining Treg cell homeostasis.

**Results:**

We isolated Treg and Th cells from a genetically diverse family of 31 BXD type recombinant inbred strains and the fully inbred parental strains of this family--C57BL/6J and DBA/2J. Subsequently genome-wide gene expression studies were performed from the isolated Treg and Th cells. A comparative analysis of the transcriptomes of these cell populations allowed us to identify many novel differentially expressed genes. Analysis of cis- and trans-expression Quantitative Trait Loci (eQTLs) highlighted common and unique regulatory mechanisms that are active in the two cell types. Trans-eQTL regions were found for the Treg functional genes *Nrp1, Stat3 *and *Ikzf4*. Analyses of the respective QTL intervals suggested several candidate genes that may be involved in regulating these genes in Treg cells. Similarly, possible candidate genes were found which may regulate the expression of *F2rl1, Ctla4, Klrb1f*. In addition, we identified a focused group of candidate genes that may be important for the maintenance of self-tolerance and the prevention of allergy.

**Conclusions:**

Variation of expression across the strains allowed us to find many novel gene-interaction networks in both T cell subsets. In addition, these two data sets enabled us to identify many differentially expressed genes and to nominate candidate genes that may have important functions for the maintenance of self-tolerance and the prevention of allergy.

## Background

Regulatory T cells (Tregs) are key modulators of immune responses in mice and humans and represent key candidates for therapeutic interventions of a broad variety of immunological diseases [1]. While reduction or functional inactivation of Tregs would be beneficial for restoration of anti-tumor immunity, selective expansion of Tregs is a promising approach for preventing autoimmunity, allergy and organ graft rejection in the transplantation setting. Initially being described as thymus-derived CD25+ subpopulation within the naïve CD4+ T-helper cell (Th) pool [2], during the last decade extensive gene expression studies based on the comparison of CD25+CD4+ Tregs and CD25-CD4+ T helper cells (Th) revealed a considerable number of additional genes critically involved in Treg development and function [3-9]. Among those, the transcription factor FOXP3 was identified as master-regulator of the Treg lineage [10-12].

Defects in the *Foxp3 *gene function in humans and mice result in fatal autoimmunity, and *Foxp3 *over-expression in previously naïve T cells converts them to Treg-like cells with *in vivo *and *in vitro *suppressive function. Despite increasing knowledge regarding the molecular signature of Tregs and mechanisms underlying their suppressive function, the extent to which Treg development and function are genetically controlled has not been studied to date.

To better understand gene variants that underlie disease predispositions related to Treg functions and to identify regulatory networks related to both Treg and Th cells, we undertook a systems genetics analysis of gene expression in these cell types using a genetic reference panel consisting of 31 members of the large BXD family of recombinant inbred strains [13,14]. Genetic reference panels (GRPs) such as the BXD family, are sets of strains that have a defined and fixed genetic architecture that can be used in classic linkage studies and complex trait analysis. The BXD family is one of the largest GRP, consisting of ~150 lines of which 80 are now fully inbred that all trace their descent from F2 progeny of crosses between C57BL/6J (B) and DBA/2J (D). Individuals within a single BXD strain are nearly isogenic (except for the sex chromosomes) and genotypes for the entire family of strains are known and stable [15]. The high level of genetic variation among BXD strains can be exploited to systematically study the genetic control of gene expression even at the level of single cell types [16] and even higher order genotype-to-phenotype relations, including for example global analysis of disease susceptibility [17-19].

Recently, whole-genome transcriptome data have been collected from GRPs. The expression level of a given transcript in a cell type or tissue may be then treated as a quantitative trait, and by employing standard linkage analyses so-called expression quantitative trait loci (eQTLs) can be identified. This links differences in expression to specific chromosomal intervals [16,20-22]. eQTL analysis can be used to identify regulatory interactions and to analyze specific effects of treatment or cell type on transcriptional control [23-27]. In addition, the analysis of eQTLs in chromosomal regions linked to disease susceptibility can help identify key candidate genes [23,28,29].

Here, we report the whole transcriptome analysis of steady-state Terg and Th cells that were isolated from 33 members of the BXD family, including the parental strains. Variation of expression across the strains allowed us to identify many novel gene-interaction networks in both T cell subsets. In addition, these two data sets enable us to nominate candidate genes that may have important functions for the maintenance of self-tolerance and the prevention of allergy.

## Results

CD4+CD25+ T regulatory (Treg) cells and CD4+CD25-T helper (Th) cells were isolated from the spleens of 31 BXD strains and the parental strains DBA/2J and C57BL/6J by fluorescence activated cell sorting. Subsequently, whole genome transcriptome analysis was performed separately for each BXD strain and for Treg and Th cells.

The gene expression data has subsequently been used for two types of analysis. (1) A direct comparison of all genes that were expressed in Treg versus Th detected was performed from all samples together to identify differentially expressed genes in either cell type. Here, the variation in expression patterns between strains was not taken into account. These studies allowed us to identify a large number of novel, Treg-specific genes. (2) Differences in gene expression patterns between strains were used to determine eQTLs in Treg and Th cells, separately. This analysis allowed us to identify cis- and trans-eQTLs, potential gene regulatory interactions and candidate genes involved in autoimmune disease.

### Analysis of differentially expressed genes reveals known and novel gene candidates important for Treg and Th functions

The large data set - for each cell type a total of 33 replicates has been generated - provided a high statistical power to identify genes that were differentially expressed in Treg versus Th cells. We identified differentially expressed genes by employing a statistical test on the difference between the two T cell populations, setting the threshold for the p-value to < 0.001 and using an expression difference of at least two-fold. In the following, probesets that exhibited a statistically significant and at least two-fold difference in expression between Treg and Th cells are referred to as 'DE-2fold genes'. In this way, a total of 14,117 probesets was found that exhibited a significantly higher expression level in Treg compared to Th cells (additional file 1, Table S1). 608 probesets were found from transcripts that were at least two-fold higher expressed in Treg cells (DE-2fold genes), including 413 annotated genes. On the other hand, using the same criteria, for Th expressed genes, a total of 11.944 probesets was found significantly higher expressed in Th compared to Treg cells, and 823 probesets, containing 481 annotated genes, showed a two-fold or higher expression level (DE-2fold genes) in Th cells (additional file 2, Table S2).

A total of 15 genes with known Treg functions (see additional file 3, Table S3) were found amongst the differentially expressed genes (additional file 3, Table S4), including well known Treg functional genes like *Foxp3 *(forkhead box P3), *Nrp1 *(neuropilin 1), *Itgae/αEβ7 *(integrin alpha E, epithelial-associated), *Ctla4 *(cytotoxic T-lymphocyte-associated protein 4), *Ikzf4/Eos *(IKAROS family zinc finger 4). *Il2ra/CD25 *(interleukin 2 receptor, alpha chain) was found to be highly expressed in Treg compared to Th cells (25-fold higher) which is expected, because the resting Treg subset was isolated on the basis of CD25 surface expression. Also, *Foxp3*, representing the most important Treg functional gene in mice, was found to be expressed 43-fold higher in Treg cells. GO term analyses for the DE genes revealed substantial differences between Tregs and Th. Whereas lymphocyte activation was indicated for Treg genes, antigen processing and presentation was strongly associated to Th genes (data not shown).

These results confirmed successful separation of highly pure Treg and Th subsets and corroborates the value of our data for subsequent analyses. Furthermore, the comparison of Treg and Th gene expression data from a large sample size allowed us to identify a large number of genes that are differentially expressed between Treg and Th cells and may play a role in the differentiation and maintenance or the function of the respective cell populations.

### Global analyses of eQTL mapping reveals different eQTLs between Treg and Th cell populations

The analysis of gene expression patterns across 31 BXD and the two parental strains allowed determining eQTLs as quantitative traits by comparing expression values for each gene and associating them with markers along the genome in all strains.

In principle, two types of eQTLs can be distinguished. If an eQTL is located at the same genomic position as the gene itself (within an operationally defined 10 Mb interval of the gene), it is considered as a cis-eQTL. In this case, variations in the promoter sequence or in regions that determine the stability of the mRNA of the gene are the most likely causes for the observed differences. If the eQTL is at a distant location (operationally defined as a distance exceeding 10 Mb) from the regulated gene, it is referred to as a trans-eQTL. For example, a transcription factor may be located in the eQTL interval which regulates the expression of the target gene. In this way, many gene regulatory regions but also gene-gene interactions and networks may be identified.

**Figure 1:**
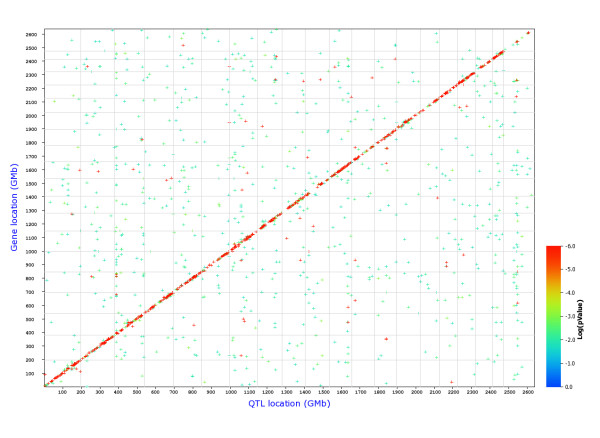
**Genome-wide graph of cis- and trans-eQTLs in Treg cells**. The positions of the eQTLs are plotted against the locations of the corresponding transcript along the genome. Cis-regulated genes are located along the diagonal, all other dots represent trans-regulated genes. The significance level of each QTL is indicated by the color. FDR = 0.3.

**Figure 2:**
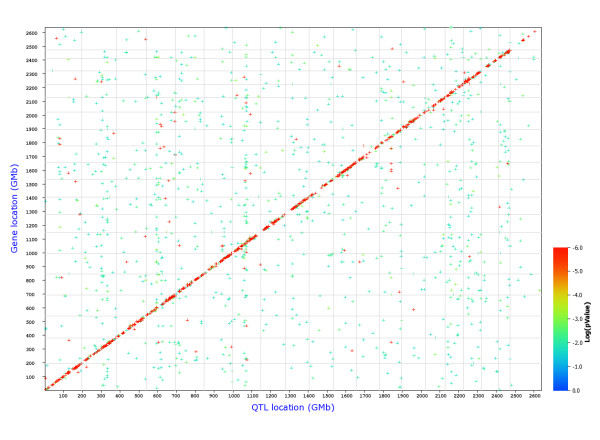
**Genome-wide graph of cis- and trans-eQTLs in Th cells**. The positions of the eQTLs are plotted against the locations of the corresponding *trans*cript along the genome. *Cis*-regulated genes are located at the diagonal, all other dots represent *trans*-regulated genes. The significance level of each QTL is indicated by the color. FDR = 0.3.

Therefore, we performed a search for e-QTLs at a global level for all probesets. Figures 1 and 2 show the position of all cis- and trans-eQTLs in Treg and Th cells, respectively. The analysis revealed that the overall distribution of trans-eQTLs between the two cell populations is different suggesting different regulatory circuits in the two cell types (see additional file 4, Figure S1 and S2 for direct comparisons of Treg and Th genome graphs).

### Many cis eQTL loci are shared between Treg and Th cells

We then identified all cis- and trans-eQTLs in both Treg and Th data sets with an LRS value of 18 or higher. An LRS of 18 for trans-eQTLs corresponds to a LOD score of 3.9 for trans-eQTLs and thus to a genome-wide p-value of about 0.05.

In the Treg data set, we identified 1,838 probesets with cis-eQTLs with LRS scores of 18 or greater. In comparison, we identified 2,153 probesets of this type in the Th data set. Of these 1,346 probesets (~62.5%) were common to both cell types.

**Figure 3:**
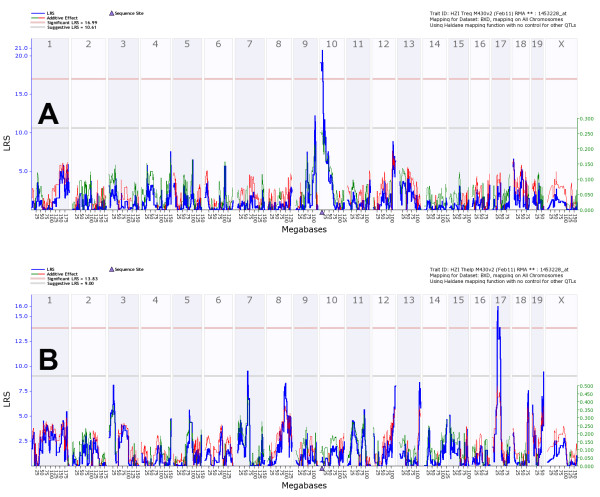
**Genome wide eQTL mapping of Stx11 transcript in Treg and Th cells**. (A) eQTL map for *Stx11 *(probeset 1453228_at) in Tregs and (B) in Th, respectively. The numbers at the top are chromosomes, and positions at the bottom are given in megabases along the chromosome. The blue line represents the significance level of the QTL expressed as LRS score (likelihood ratio statistic). A positive additive coefficient (green line) indicates that DBA/2J alleles increased trait values. A negative additive coefficient (red line) indicates that C57BL/6J alleles increased trait values. The two horizontal lines mark the genome-wide significance levels at p < 0.05 (red line) and p < 0.37 (gray line). A blue triangle marks the position of the gene.

Sixty-seven of the cis-eQTL probesets in Tregs also represented DE-2fold genes for Tregs. Despite the large overlap of cisQTL in the Th and Treg samples, 35 probesets (52%) were only found in Treg samples and were not detected as cis-eQTLs in Th using the above criteria of a minimum LRS of 18 and DE-2fold for Treg (additional file 3, Table S5). However, it should be noted that some probesets, e.g. *Lad1 *(ladinin) *Naip5/Birc1e *(NLR family, apoptosis inhibitory protein 5) and *Gbp4 *(guanylate binding protein 4)) also exhibited significant cis-eQTLs in Th but the corresponding LRS did not reach the threshold level of 18 (data not shown). Figure 3 shows *Stx11 *(syntaxin 11) as an example for a gene that was expressed both in Treg and Th cells but exhibited a strong cis-eQTL only in Treg cells. *Stx11, Gsta4, Ctram, Zfp467 *exhibited a cis-eQTL in Treg and a trans-eQTL in Th cells.

In the Th data set, 70 of the identified cis-eQTLs in Th were also DE-2fold genes for Th cells. Of these, 35 probesets represented cis-eQTL genes that were only found in Th cells and were not detected as cis-eQTLs with a LRS of 18 or larger inTreg (additional file 3, Table S6). However, some probestes also exhibited strong cis-eQTLs in Treg but the corresponding LRS did not reach the threshold level of 18, e.g. *Snx5 *(sorting nexin 5, probeset 1417646_a_at) exhibited a significant cis-eQTL in Th and a strong trans-eQTL in Treg cells (data not shown). Similarly, *Chn2, Cadm1 *and *Themis/E430004N04Rik *showed a cis-eQTL in Th and a trans e-QTL in Treg cells.

Strong cis-eQTLs highlight genes that are differentially expressed from the two parental alleles. They can be employed in a forward genetic approach in subsequent studies to elucidate the biological function of the respective genes.

### Trans-eQTL mapping reveals different mechanisms of gene regulation in Treg and Th cells

We then analyzed trans-eQTLs at a global level, using again a LRS threshold of 18 or higher. 1,019 probesets were identified which showed a trans-eQTLs in Treg cells. Of these, 65 probesets exhibited trans-eQTLs that were present both in Treg and Th cells. 25 probesets showed also a two-fold or higher level of expression (DE-2fold genes) in Treg cells (additional file 3, Table S7). Of these, all except *BC010605 *and *Prdm5*, represented Treg-specifc trans-eQTLs (see M&M for definition of Treg specific trans-eQTLs).

**Figure 4:**
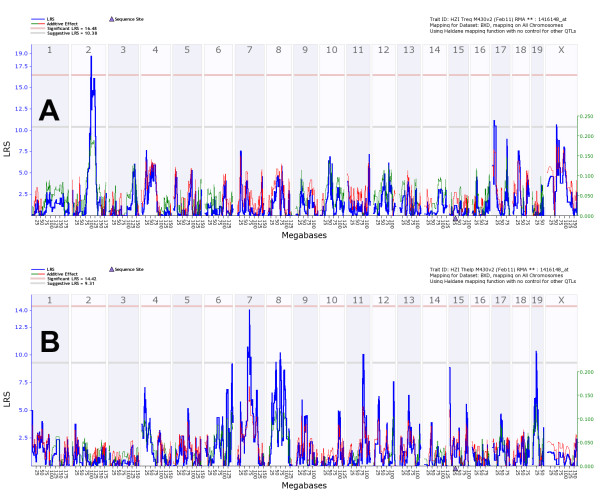
**Genome wide eQTL mapping of Laptm4b transcript in Treg and Th cells**. (A) eQTL map for *Laptm4b *(probeset 1416148_at in Tregs and (B) in Th, respectively. See figure 3 for explanation of labels.

For *Abcb1a *(ATP-binding cassette, sub-family B (MDR/TAP), member 1A), *Gata1 *(GATA binding protein 1), *Mapkbp1 *(mitogen-activated protein kinase binding protein 1), *Marco *(macrophage receptor with collagenous structure), and *Nr4a2/Nurr1 *(nuclear receptor subfamily 4, group A, member 2) an immune-related function, in some cases with regulating activity, has been described (see discussion). *Laptm4b *(lysosomal-associated protein transmembrane 4B) and *Lycat *(lysocardiolipin acyltransferase 1) exhibited strong trans-eQTL signals in both cell types but at different chromosomal locations (Figures 4 and S3, respectively).

In the Th data set, 1,099 probesets were identified exhibiting a trans-eQTLs of 18 or higher. Of these, 30 probesets showed a two-fold or higher level of expression (DE-2fold genes) in Th cells (additional file 3, Table S8) and all, except *BC018101 *and *Prpf3*, represented Th-specifc trans-eQTLs.

The expression of *Prpf3 *(PRP3 pre-mRNA processing factor 3 homolog (yeast)) showed strong and shared trans-eQTLs in Th and Treg cells (additional file 4, Figure S4), whereas *St8sia1 *(ST8 alpha-N-acetyl-neuraminide alpha-2,8-sialyltransferase 1), *Crtam *(cytotoxic and regulatory T cell molecule), *Igfbp4 *(insulin-like growth factor binding protein 4) and *Sell *(selectin, lymphocyte) exhibited strong trans-eQTL signals in both cell types but at different chromosomal locations (data not shown).

In summary, the identification of cell-specific trans-eQTL regions allowed the subsequent analyses of candidates in the respective regions and to propose novel gene-gene regulatory interactions (see below).

### QTLs analysis for known functional Treg genes shows trans-regulation for Nrp1 in Treg

In order to identify gene interaction networks for genes that may play an important role for Treg function, we studied known Treg functional genes (see additional file 3, Table S3) that were differentially expressed in Treg cells and that showed a high expression signal in Tregs (expression signal > 8). This analysis revealed four genes with eQTLs of an LRS equal or larger than 14 (additional file 3, Table S9): *Tnfrsf1b *(tumor necrosis factor receptor superfamily, member 1b), *Nrp1 *(neuropilin 1), *Stat3 *(signal transducer and activator of transcription 3) and *Ikzf4 *(IKAROS family zinc finger 4). *Nrp1 *(additional file 4, Figure S5) and *Tnfrsf1b *(data not shown) exhibited a genome-wide significant trans-eQTL, whereas the eQTLs for *Stat3 *and *Ikzf4 *did not reach genome-wide significance levels. Also, *Foxp3 *and *Ctla4 *did not exhibit any significant cis- or trans-eQTLs in Treg cells.

These trans-eQTL regions were subsequently used to propose novel gene-gene regulatory interactions (see below).

### Analysis of candidate genes in trans-eQTL intervals reveals novel gene-gene regulatory interactions and relation to classical phenotype traits

Next, we analyzed several trans-eQTLs that were identified in the above analyses for candidate genes that may be involved in the regulation of target gene expression (additional file 3, Table S10). Below, we present the analysis of one QTL interval as an example. The results for other QTL intervals can be found in additional file 5, 'Additional Results and Discussion.

The interval on chromosome 4 (28 - 35 Mb) was found to regulate expression of the *F2rl1 *gene (additional file 4, Figure S6). This region contains 37 probesets which were well expressed (> 8) in Treg cells (Table S11). Of these, *Ccdc111 *(coiled-coil domain containing 111), *Zfp292 *(zinc finger protein 292), *1810030N24Rik, AI448984 *and *Map3k7 *(mitogen-activated protein kinase kinase kinase 7) exhibited strong cis-eQTLs (LRS > = 15) and thus are most likely candidates for regulating the expression of *F2rl1 *in Treg cells.

Furthermore, we analyzed the same eQTL intervals for the presence of a significant QTL in classical phenotypes that are contained in the GeneNetwork database. A total of 18 phenotypes could be found with an LRS of 18 or larger. Of these, eight were related to immune and infectious diseases traits (Table 1). Thus, the candidate genes may also be involved in regulating these traits. It should, however, be noted that our search algorithm only detects classical phenotypes that exhibit a peak QTL at this position. A more detailed analysis can be performed manually for any phenotype of interest.

**Table 1:** QTLs from classical phenotypes overlapping with eQTLs

Chromosome	Mb	Record	Phenotype	Max LRS	Max LRS Location (Chr: Mb)
3	20-30	12958	Blood chemistry, cardiovascular system: White blood cell count of 14-week old males	19	Chr3: 28.293133
4	28-35	10410	Immune function: Antigenic activity of irradiated BXD spleen cells for Thy1+CD3+CD4+CD8- T-cell clone TGVH32	31	Chr4: 31.651725
17	25-45	11025	Infectious disease, immune function: Chlamydia psittaci (6BC) infection response (10^4 IFU ip), pathogen load in peritoneal cavity at 30 days among surviving 3-5 month males	33.6	Chr17: 31.769795
17	25-45	10466	Immune function, gastrointestinal system: Intestinal intraepithelial gamma-delta type T lymphocytes (i-IELs) in 2-6 month old adults based on receptor expression (V-gamma-7 positive, Vgamma-4 negative T cells)	33.1	Chr17: 32.068297
17	25-45	10236	Proliferation of JTL-G12 cells (T cell clone) with 50 ug/ml GAT (Glu60, Ala30, Tyr10)	24.5	Chr17: 32.068297
17	25-45	10238	Proliferation of JTL-G12.8 (Tcell clone) with 50 ug/ml GAT (Glu60, Ala30, Tyr10)	26.7	Chr17: 32.068297
17	25-45	10441	Anti-F antigen (liver protein) titer, experiment 1	19.1	Chr17: 26.950946
17	25-45	12672	Infectious disease, immune function: Ectromelia virus survival over two weeks after a 90 pfu intranasal inoculation, males and females between 40 and 150 days of age, residuals corrected for sex, age, and body weight	19.5	Chr17: 31.179359

### Analysis of autoimmune traits

In a previous study for autoimmune disease traits, two QTLs were identifies that are associated with autoimmune traits in the BXD population. One QTL on chromosome 2 was associated with elevated serum titers of anti-DNA antibodies and a second locus on chromosome 4 with increased serum titers of rheumatoid factor (RF) levels [30]. Therefore, we investigated these intervals in our Treg and Th data sets.

The QTL interval (150 - 170 Mb) on chromosome 2 contains 286 probesets that were expressed in Treg cells (signal > 8) and 290 probesets were expressed in Th cells (signal > 8), respectively. We then identified the genes that exhibit a cis-eQTL in Treg cells because these may be involved in the regulation of autoimmunity to DNA. 19 probesets were identified that also exhibited a strong cis-eQTLs (LRS > = 15) in Treg cells (additional file 3, Table S12). The peak interval (160 - 165 Mb) contained the genes *Ada *(adenosine deaminase), *Slpi *(secretory leukocyte peptidase inhibitor), *Sys1/2610042O14Rik *(SYS1 Golgi-localized integral membrane protein homolog (*S. cerevisiae*)), and *Znf335 *(zinc finger protein 335). The cis-eQTL signal for *Ada *is most likely caused by SNPs in the hybridization probe. Thus these genes, with the exception of *Ada*, represent likely candidates for regulating this trait in Treg cells. The results for the analysis of the chromosome 4 interval can be found in the supplement data.

## Discussion

Here, we studied the expression patterns of genes in Treg and Th cells from a large number of individuals from the BXD recombinant inbred strain collection. The ultimate goal of these studies was the identification of expression quantitative traits (eQTLs) and of co-regulated genes that will give new insights into gene-gene interactions and regulatory pathways. In addition, the large data set of expression profiles from 33 mouse lines allowed us to validate known and to newly identify so far unknown genes that are expressed specifically in Treg but not Th cells.

Until now, several studies have aimed to identify genes that are specific for Treg cells (additional file 3, Table S3). Using our data set from 33 mouse stains allowed us to search for differences in expression levels in Treg versus Th cells with a so far unprecedented statistical power. Our analysis confirmed that many genes with a described Treg function are also differentially expressed. Of the 23 genes that were described in the literature as genes with an important function in Treg cells, 17 were also found to be Treg specific in our analysis. Of the remaining six genes, Galectin-10 does not have a homologue in humans. *S1pr1a *and *Lrrc32 *were not represented on the array. Also a Treg function for LRRC32/GARP was so far only described in humans. The remaining three genes, *Gzmb, Lag3 *and *Il12rb2*, were expressed at low levels in both Treg and Th but with no significant difference between the two cell types.

The genes *Foxp3, Nrp1, Clta4, Tnfrsf18 */*Gitr, Il2ra */*Cd25, Itgae */*CD103, Ikzf4 */*Eos, Gpr83 *have been reported to be hallmark genes for Treg cells [31,32]. Indeed, all of them appear as DE-2fold genes specifically higher expressed in Treg cells. Since we discovered *Nrp1 *as surface marker of murine regulatory T cells [3] comprehensive data have been obtained regarding its implications in Treg function [33-37]. Whereas expression of other Treg-markers such as *Cd25 *and *Ctla4 *are induced upon activation of Th cells, activated murine Th cells lack *Nrp1 *expression, suggesting fundamental differences in the transcriptional regulation of this molecule in Tregs when compared with other well described Treg markers.

In total, 608 probesets were found in our analysis as Treg-specific and with an expression difference of at least two-fold. Although a difference of two-fold in expression levels is often used in gene expression array studies in many studies, it may be considered as arbitrary. Our study allowed to find even more genes with statistical significant differences in expression levels and extended the known list to 14,117 probesets that are specific for differentiated Treg versus Th cells. Zheng et al. [38] performed chromatin immunoprecipitation and genome tiling array profiling to identify direct targets of the *Foxp3 *transcription factor. About 270 genes were identified were also up-regulated in Foxp3^+ ^T cells. Of these, 162 genes were also found as differentially expressed genes in our analysis.

Recently, a genome wide association study in a large cohort of patients suffering from alopecia areata, which is among the most highly prevalent human autoimmune diseases, leading to disfiguring hair loss due to the collapse of immune privilege of the hair follicle and subsequent autoimmune attack revealed an association with genomic regions containing several genes controlling the activation and proliferation of Tregs including *Ctla4, CD25*, and *Eos/Ikzf4 *[39].

In conclusion, almost all genes described previously as Treg functional genes or in other screening assays were also found in our analysis. These findings confirm that our study is well suitable to identify genes that are important for Treg function, differentiation and/or maintenance of their differentiation status.

Cis-eQTL genes most likely carry a mutation in the promoter region or contain a structural variation in the transcribed region which results in low expression levels or unstable RNA of one but not the other allele. In our study, several cis-eQTL genes were found that were active both in Treg and Th cells and have a known function in the immune response. For example, *Stx11 *(syntaxin 11) has been associated with familial hemophagocytic lymphohistiocytosis 4 in humans [40,41].

BXD strains that carry a very low expressing allele inherited from one of the parental strains are similar to genetic knock-down experiments or hypomorphic mutants [42]. The function of such genes can be studied in a reverse genetics approach by comparing the mean phenotypes of BXD strains that have inherited either the low or high allele--a method referred to as reverse complex trait analysis. Prominent examples of genes that are nature "knock-downs" in the BXD strains, include *Hc, Ahr, Gpnmb, Tyrp1, Sae1, Apoa2*, and several CLEC and KLRA gene family members [23,42].

341 probesets represented cis-eQTLs shared by several tissues, in Treg and Th cells but also in brain, kidney and lung.

In Treg cells, 25 probeset were trans-regulated and represented genes that were differently expressed between Treg and Th cells and that showed a two-fold or higher level of expression (DE-2fold genes) in Treg cells. Several of them represented genes with a known immune-related function. For example, *Abcb1a *(ATP-binding cassette, sub-family B (MDR/TAP), member 1A) is involved in the down-modulation of dendritic cell functions through the regulation of pro-inflammatory cytokine secretion [43]. *Gata1 *(GATA binding protein 1) has been shown to be an important regulator of mast cell differentiation [44]. *Mapkbp1 *(mitogen-activated protein kinase binding protein 1) encodes a protein that enhances NF-kappaB activation induced by MAP kinase kinase kinase 7 and TNF Receptor-Associated Factor 2 [45]. *Marco *(macrophage receptor with collagenous structure) exhibits multiple functions in the innate immune response. MARCO, TLR2, and CD14 are required for macrophage cytokine responses to mycobacterial trehalose dimycolate and Mycobacterium tuberculosis [46]. MARCO-deficient mice exhibit lower IL-12 production in responses to stimulation [47]. A defect in *Marco *results in an impaired clearance of apoptotic cells and a generalized defect in both endocytosis and phagocytosis [48]. Expression of MARCO is required for TLR signaling [46]. But Marco also exhibits suppressive functions by decreasing inflammation in lungs after exposure to ozone [49]. *Nr4a2/Nurr1 *(nuclear receptor subfamily 4, group A, member 2) represents a transcriptional mediator of inflammatory signals [50,51]. Also, it plays an important role in modulating IL-8 expression [52].

Two genes, *Laptm4b *and *Lycat *exhibited strong trans-eQTL signals in both cell types but at different chromosomal locations. Thus, the same genes are expressed in both cell types, but are likely regulated by different mechanisms. *Laptm4b *(lysosomal-associated protein transmembrane 4B) is involved in cell proliferation and multidrug resistance [53], whereas no biological function has yet been described for *Lycat *(lysocardiolipin acyltransferase 1).

None of these genes represents a known Treg key gene. Our analysis thus allows expanding the list of genes with potentially important functions in Treg cells. We did not perform an extensive analysis for trans-eQTLs in Th cells but a similar result can be expected for Th cells.

We then analyzed several trans-eQTLs for potential regulator genes in order to propose possible novel gene-regulatory circuits in Treg and Th cells. These are by far not comprehensive but rather serve as examples to illustrate how our data set will allow searching for possible regulatory networks.

A QTL region was found on chromosome 4 that regulates the expression of the *F2rl1 *(coagulation factor II (thrombin) receptor-like 1) gene. *F2rl1/Par2 *gene expression has been associated with the activation and suppression of inflammatory responses. Overexpression of *F2rl1 *in allergic inflammation of the airway exacerbates eosinophil infiltration into the lumen and hyperreactivity of the airway, while *F2rl1 *deletion diminishes inflammatory cell infiltration and reduces hyperreactivity [54]. Also, *F2rl1 *plays a protective role during influenza virus type A infection through IFN-gamma production and decreased excessive recruitment of inflammatory cells to lung alveoli [55] and deletion of *F2rl1 *is associated with decreased clearance of *P. aeruginosa *[56]. Several candidate genes in the chromosome 4 QTL interval, which regulates *F2rl1 *expression, exhibited a cis-eQTL and thus represent potential regulators of *F2rl1 *(Table S11). These include the *Map3k7/Tak1 *(mitogen-activated protein kinase kinase kinase 7) protein kinase gene which exhibits many immune modulator functions. It mediates activation of IKK (inhibitor of kappaB kinase) and silencing of Map3k7 suppressed T cell receptor-dependent IKK activation and interleukin-2 production in T cells [57,58]. The *Zfp292 *(zinc finger protein 292) gene expression has been shown tocorrelate with growth hormone expression and may thus be involved in mediating proliferation signals [59]. The analysis of candidate genes in the other QTL regions is discussed in the supplement data.

Our analysis of the chromosome 2 regions autoimmune trait [30] identified several loci that exhibited a cis-eQTL in Treg cells and may thus be involved in regulating this trait: *Slpi, Sys1/2610042O14Rik*, and *Znf335*. *Slpi *(secretory leukocyte peptidase inhibitor) exhibits many biological functions, including anti-bacterial, anti-fungal, anti-viral, anti-inflammatory, wound healing and immuno-modulatory activities [60-68]. The Slpi protein represents a ligand for PLSCR1 and PLSCR4, which interact directly with the CD4 receptor at the cell surface of T lymphocytes [69]. *Slpi *is a prominent innate immune protein of the respiratory tract with serine protease inhibitor activity [70]. It attenuates excessive inflammatory responses resulting in a balanced innate immunity [71,72]. Constitutive expression of *Slpi *reduced the inflammatory response and improved lung function in an acute model of allergic asthma in *Slpi *transgenic and knockout mice [73]. The attenuation of the inflammatory response by *Slpi *is mediated through macrophages that secrete an increased amount of Slpi when encountering apoptotic cells [74,75]. *Znf335 *(zinc finger protein 335, also *NIF1 *in human) is a cotransducer that regulates the activity of the nuclear hormone receptor coactivator NRC [76] which may mediate the function of the CCR4 signal [77]. No biological functions were identified so far for *Sys1 *(SYS1 Golgi-localized integral membrane protein homolog (*S. cerevisiae*)). The discussion of candidate genes in the chromosome 4 auto-immune QTL interval can be found in the supplemental data.

## Conclusions

Our study revealed many novel Treg specific genes and also points to genes that may regulate important gene-gene interactions in Treg cells. These interactions, once validated, should allow proposing new targets for therapeutic interventions in cancer, auto-immunity, and allergies.

## Methods

### Mouse strains and sample preparation

Parental and BXD lines were received from Jackson Laboratory, or from The Oak Ridge National Laboratory (BXD43, BXD51, BXD61, BXD62, BXD65, BXD68, BXD69, BXD73, BXD75, BXD87, BXD90), and were bred in the facility of the Neuro-BSIK consortium (VU University Amsterdam). Mice were housed on sawdust in standard Makrolon type II cages with food (Harlan Teklad 2018) and water ad libitum under specific pathogen free conditions. For the analysis, mice were transferred to the animal facility in Braunschweig and adapted for at least two weeks to the new environment before preparing the spleen cells. All protocols involving mice were approved by national animal welfare committees.

### Sorting of Tregs and Th cells by fluorescent activated cell sorting

For sorting of Tregs and Th cells, splenocytes from 31 BXD recombinant inbred strains as well as from the parental mouse lines DBA/2J and C57BL/6J were isolated by flushing the spleens with erythrocyte-lysis-buffer. Cells were collected by centrifugation, re-suspended in cold FACS-buffer (PBS/2% FCS/0.5 mM EDTA). For each strain and sex, the cells from three individual mice were pooled. After passing the cells through a 100 μm cell strainer and an additional washing step with FACS-buffer, splenocytes were stained with anti-CD4-APC and anti-CD25-PE (BD Biosciences (Heidelberg, Germany)) for 10 minutes at 4°C, washed and re-suspended in FACS-buffer. CD4+ T cells were separated into CD4+CD25+ Treg and CD4+CD25- Th cells using a MoFlo cell sorter (Cytomation) and purity of the sorted T cell subsets reached 95-97%.

### Microarray analysis

Quality and integrity of the total RNA isolated from 1 × 10^5 ^cells was controlled by running all samples on an Agilent Technologies 2100 Bioanalyzer (Agilent Technologies; Waldbronn, Germany). RNA amplification and labelling was done according to manufactures protocol (Small Sample Target Labeling Assay Version II, Affymetrix; Santa Clara, CA). The concentration of biotin-labeled cRNA was determined by UV absorbance. In all cases, 10 μg of each biotinylated cRNA preparation were fragmented and placed in a hybridization cocktail containing four biotinylated hybridization controls (BioB, BioC, BioD, and Cre) as recommended by the manufacturer. Samples were hybridized to an identical lot of Affymetrix MOE430 2.0 for 16 hours at 46°C. After hybridisation the GeneChips were washed and stained using the Affymetrix's recommended EukGE-WS2v5 protocol for GeneChip^® ^Fluidics FS400 station. Images were scanned using GeneChip^® ^Scanner 3000 under the control of GCOS 1.3 software package (Affymetrix; Santa Clara, CA)

### Data preprocessing and analysis

Microarray data was preprocessed using the RMA method [78] and log_2 _values were computed. The Z scores for each cell value was then calculated (including subtraction of means and division by standard deviations), multiplied by 2 and a value of 8 was added. The advantage of this variant of a Z transformation is that all values are positive and that 1 unit represents approximately a 2-fold difference in expression as determined using the spike-in control probesets (see [26] for details). The data was then batch corrected [79]. In this study, RNA was extracted at three different points in time for the Treg samples and also microarray processing was performed at three different points in time. Similarly, the Th samples were processed in two batches. Therefore, we performed a batch correction for both cell types using the following ANOVA model before further analysis of the data: *y_i _= μ + B_i _+ e_i _*where *y_i _*is the expression level of the *i*^th ^microarray, *μ *is the overall mean, B*_i _*is the batch to which the *i*^th ^individual belongs and *e_i _*is the residual error. Finally, mean values were calculated if multiple samples from one BXD line were recorded (male and females or replicates) and the processed data were stored and are publically available in the GeneNetwork (GN) database [20]. Identification and QTL analyses were performed using the GeneNetwork web service [20] as previously described [23].

Differentially expressed probes between Treg and Th were calculated by applying the moderated F-statistic [80], an empirical Bayes method, Cut-offs were set to pFDR < = 0.001 and fold change > = 2×. Probes significantly higher expressed either in Treg or Th, respectively, were tested for statistically associated GO terms via the hypergeometric test. Data analyses were done in the R environment with Bioconductor [81] and packages limma [78] and GOstats [82].

The GeneRIF database http://www.ncbi.nlm.nih.gov/projects/GeneRIF/GeneRIFhelp.html was used as a primary source to search for known gene functions and to cite these in the discussion. We also tested whether a trans-eQTLs obtained in one data set is specific for this cell type. GeneNetwork does not have an automatic function for such an analysis. We therefore tested this feature manually in the following way. For trans-eQTLs with an LRS of 18 or higher in Treg cells, the LRS was determined at the same marker positions in the Th data set. If the LRS in Th was less that 6 (which corresponds to a p-value of less than 0.05 at this marker - not a genome-wide significance) the QTL in Treg was considered "Treg-specific". The analysis was performed likewise to determine Th-specifc trans-eQTLs.

### Access to microarray data

The processed data and analysis tools are available at the GeneNetwork database http://www.genenetwork.org/webqtl/main.py, accession number for Treg data: GN122; accession number for Th data: GN319. The raw and processed data are accessible from the ArrayExpress database, ID: E-MTAB-836.

## Competing interests

The authors declare that they have no competing interests.

## Authors' contributions

DB, HC and KS generated and analyzed the material. RA, KS and RW performed the analysis of the data. GS and SS provided the mice for the analysis. DB and KS wrote the manuscript. CP performed the identification of DE genes and GO term analyses. All authors read and approved the final manuscript.

## Supplementary Material

Additional file 1 **Table S1**. Treg specific genes. The table lists genes that exhibited a differential expression in Treg compared to Th cells. Probeset: probeset ID of microarray hybridization probe, logFC: fold changes of expression level in Treg versus Th as difference of log_2 _values, pFDR: FDR-corrected p-value, Treg: expression level in Treg cells as log_2 _value, Th: expression level in Th cells as log_2 _value. Click here for file

Additional file 2 **Table S2 Th specific genes**. The table lists genes that exhibited a differential expression in Th compared to Treg cells. Probeset: probeset ID of microarray hybridization probe, logFC: fold changes of expression level in Treg versus Th as difference of log_2 _values (a negative value represents higher expression in Th compared to Treg cells), pFDR: FDR-corrected p-value, tr: expression level in Treg cells as log_2 _value, th: expression level in Th cells as log_2 _value. Click here for file

Additional file 3 **Table S3 Genes with known functions in Treg cells**. The table lists genes that have been described in the literature to exhibit a functional role in Treg cells. **Table S4: Treg functional genes with a higher expression in Tregs.** The table lists probesets of genes that were expressed at a higher level in Treg compared to Th cells and which exhibit known Treg functions. **Table S5. Genes regulated by cis-eQTL in Treg cells.** The table lists probesets from genes that exhibited a cis-eQTL larger or equal to an LRS of 18 in Treg but not in Th cells and which were expressed at least 2-fold higher in Treg cells. **Table S6. Genes regulated by cis-eQTL (LRS > = 18) in Th.** The table lists probesets from genes that exhibited a cis-eQTL larger or equal to an LRS of 18 in Th but not in Treg cells and which were expressed at least 2-fold higher in Th cells. **Table S7. Treg specific genes regulated by trans-eQTL in Treg cells. **The table lists probesets from genes that exhibited a trans-eQTL larger or equal to an LRS of 18 in Treg but not in Th cells and which were expressed at least 2-fold higher in Treg cells. **Table S8. Th specific genes regulated by trans-eQTL in Th cells.** The table lists probesets from genes that exhibited a trans-eQTL larger or equal to an LRS of 18 in Th but not in Treg cells and which were expressed at least 2-fold higher in Th cells. **Table S9. Treg functional genes regulated and highly expressed in Treg cells. **The table lists probesets of differentially expressed genes in Treg cells with a known Treg function, a high expression signal in Tregs (expression signal > 8) and which are regulated by a trans-eQTL with an LRS > = 14. **Table S10: Intervals selected for further QTL analysis.** The table lists the QTL intervals from selected Treg and Th cis- and trans-eQTLs that were further analyzed for the presence of candidate regulatory genes. **Table S11 Candidate genes located in the *F2rl1-*QTL interval on chromosome 4. **The table lists probesets of possible candidate genes that are located in the QTL interval on chromosome 4 which regulates the expression of *F2rl1*. Only genes with an expression signal larger than 8 were selected. **Table S12 Candidate genes located in the QTL interval on chromosome 2 (autoimmunity to DNA).** The table lists probesets of possible candidate genes that are located in the QTL interval on chromosome 2 which regulates autoimmunity to DNA. Only genes expressed in Treg cells (signal > 8 on log_2 _scale) and exhibiting a cis-eQTL of LRS > = 15 were selected. Click here for file

Additional file 4 **Figure S1**. Genome-wide graph of cis- and trans-eQTLs in Treg and Th cells. The file shows the genome-wide mapping graphs for Treg cells and the same graph for Th cells overlaid in grey. **Figure S2. Genome-wide graph of cis- and trans-eQTLs in Th and Treg cells.** The file shows the genome-wide mapping graphs for Th cells and the same graph for Treg cells overlaid in grey. **Figure S3, Genome wide eQTL mapping of *Lycat *transcript in Treg and Th cells.** The file contains a graph showing the result of a genome-wide mapping of eQTLs for the *Lycat *transcript in Treg and Th cells. **Figure S4, Genome wide eQTL mapping of *Prpf3 *transcript in Treg and Th cells.** The file contains a graph showing the result of a genome-wide mapping of eQTLs for the *Prpf3 *transcript in Treg and Th cells. **Figure S5, Genome wide eQTL mapping of *Nrp1 *transcript in Treg and Th cells. **The file contains a graph showing the result of a genome-wide mapping of eQTLs for the *Nrp1 *transcript in Treg and Th cells. **Figure S6, Genome wide eQTL mapping of *F2rl1 *transcript in Treg and Th cells.** The file contains a graph showing the result of a genome-wide mapping of eQTLs for the *F2rl1 *transcript in Treg and Th cells. **Figure S7, Genome wide eQTL mapping of *Ctla4 *transcript in Treg and Th cells.** The file contains a graph showing the result of a genome-wide mapping of eQTLs for the *Ctla4 *transcript in Treg and Th cells. Click here for file

Additional file 5 **'Additional Results and Discussion': This file contains additional results on: Trans-eQTLs mapping in Treg and Th cells, the analysis of candidate genes in trans-eQTL intervals, and the analysis of autoimmune traits**. Also, the file contains additional discussion points on Treg-specifc genes, the analysis of the interval on chromosome 2 found to regulate expression of *Nrp1*, analysis of an interval on chromosome × regulating expression of *Klrb1f/A630024B12Rik *(killer cell lectin-like receptor subfamily B member 1F) and *Ctla4 *(cytotoxic T-lymphocyte-associated protein 4), the analysis of known Treg functional genes for possible cis- or trans-eQTLs, and the analysis of the QTLs interval chromosome 4 associated with autoimmune traits. Click here for file
