# Supplementary material for: Expression QTL mapping in regulatory and helper T cells from the BXD family of strains reveals novel cell-specific genes, gene-gene interactions and candidate genes for auto-immune disease
Source: BMC Genomics. 2011 Dec 19;12:610. doi: 10.1186/1471-2164-12-610 (PMC3277499; doi:10.1186/1471-2164-12-610)
Supplement: Additional file 3 — Table S3 Genes with known functions in Treg cells. The table lists genes that have been described in the literature to exhibit a functional role in Treg cells. Table S4: Treg functional genes with a higher expression in Tregs. The table lists probesets of genes that were expressed at a higher level in Treg compared to Th cells and which exhibit known Treg functions. Table S5. Genes regulated by cis-eQTL in Treg cells. The table lists probesets from genes that exhibited a cis-eQTL larger or equal to an LRS of 18 in Treg but not in Th cells and which were expressed at least 2-fold higher in Treg cells. Table S6. Genes regulated by cis-eQTL (LRS > = 18) in Th. The table lists probesets from genes that exhibited a cis-eQTL larger or equal to an LRS of 18 in Th but not in Treg cells and which were expressed at least 2-fold higher in Th cells. Table S7. Treg specific genes regulated by trans-eQTL in Treg cells. The table lists probesets from genes that exhibited a trans-eQTL larger or equal to an LRS of 18 in Treg but not in Th cells and which were expressed at least 2-fold higher in Treg cells. Table S8. Th specific genes regulated by trans-eQTL in Th cells. The table lists probesets from genes that exhibited a trans-eQTL larger or equal to an LRS of 18 in Th but not in Treg cells and which were expressed at least 2-fold higher in Th cells. Table S9. Treg functional genes regulated and highly expressed in Treg cells. The table lists probesets of differentially expressed genes in Treg cells with a known Treg function, a high expression signal in Tregs (expression signal > 8) and which are regulated by a trans-eQTL with an LRS > = 14. Table S10: Intervals selected for further QTL analysis. The table lists the QTL intervals from selected Treg and Th cis- and trans-eQTLs that were further analyzed for the presence of candidate regulatory genes. Table S11 Candidate genes located in the F2rl1-QTL interval on chromosome 4. The table lists probesets of possible candidate genes t [file 1471-2164-12-610-S3.PDF]

Table S3 Genes with known functions in Treg cells

Mouse gene name	Other synonyms	Reference
Foxp3		[1]
Il2ra	CD25	[2]
Gpr83		[3]
Nrp1	Nrp	[4]
Ikzf4	Eos	[5]
Tnfrsf18	Gitr	[6]
Ebi3	IL35	[7]
Ctla4		[8]
S1pr1	S1p1	[9]
Gzmb		[10]
Lgals1	Galectin-1	[11]
Itgae	CD103	[12]
Lag3	CD223	[13]
Entpd1	CD39	[14, 15]
Stat3		[16]
Irf4	Irf4	[17, 18]
Tnfrsf1b	Tnfr2	[19]
Runx3	Runx3	[20]
Gja1	Cx43	[21]
Phlpp1	Phlpp1	[22]
Prdm1	Blimp-1	[18]
Lrrc32	GARP, human only	[23, 24]
Il1r1	IL1R1, human only	[25]
Il1r2	IL1R2, human only	[25]
Clc	GALECTIN-10, human only	[26]
Smad3	SMAD3, human only	[27]
Nfat5	NFAT5, human only	[27]

Table S4 List of probesets with a higher expression in Tregs compared to Th cells and with known Treg functions.

Probeset	Gene Name	logFC	Treg	Th
1420765_a_at	*Foxp3*	5.436	12.4	6.9
1420692_at	*Il2ra*	4.652	11.5	6.9
1447541_s_at	*Itgae*	4.468	12.2	7.7
1419334_at	*Ctla4*	3.420	13.2	9.8
1449216_at	*Itgae*	3.290	11.1	7.8
1439569_at	*Gpr83*	2.913	12.6	9.7
1418084_at	*Nrp1*	2.802	12.8	10.0
1438274_at	*Ikzf4*	2.630	11.3	8.7
1457342_at	*Ikzf4*	2.541	11.9	9.4
1423415_at	*Gpr83*	2.482	12.2	9.7
1420691_at	*Il2ra*	2.381	9.5	7.1
1448944_at	*Nrp1*	2.166	11.5	9.3
1426994_at	*Phlpp1*	2.056	11.4	9.3
1448943_at	*Nrp1*	2.018	13.2	11.2
1449222_at	*Ebi3*	1.971	11.5	9.5
1418099_at	*Tnfrsf1b*	1.895	11.5	9.6
1421173_at	*Irf4*	1.808	10.7	8.9
1422303_a_at	*Tnfrsf18*	1.804	12.5	10.7
1448950_at	*Il1r1*	1.205	9.4	8.2
1419532_at	*Il1r2*	1.103	9.7	8.6
1420425_at	*Prdm1*	1.027	9.5	8.5
1448951_at	*Tnfrsf1b*	0.878	8.6	7.8
1453586_at	*Entpd1*	0.805	8.2	7.4
1460664_at	*Ikzf4*	0.773	7.3	6.5
1460700_at	*Stat3*	0.585	11.4	10.9
1426587_a_at	*Stat3*	0.564	10.8	10.3
1423326_at	*Entpd1*	0.513	8.7	8.2
1415801_at	*Gja1*	0.493	7.0	6.5
1455439_a_at	*Lgals1*	0.395	13.8	13.4
1419573_a_at	*Lgals1*	0.387	12.7	12.3

1451921_a_at	*Nfat5*	0.348	6.5	6.1
1438999_a_at	*Nfat5*	0.347	10.0	9.6
1454960_at	*Smad3*	0.338	11.2	10.9
1450472_s_at	*Smad3*	0.286	7.2	6.9
1438973_x_at	*Gja1*	0.284	6.3	6.0
1421467_at	*Runx3*	0.135	7.5	7.3

Expression levels from 31 BXD strains and parental strains were determined for Treg and Th cells. Subsequently, all probsets that exhibited a significantly higher expression level in Treg compared to Th cells (p<= 0.001) were identified and the genes with known Treg functions were selected. Probeset: probeset ID of microarray hybridization probe, logFC: fold change of expression level in Treg versus Th as difference of log_2 values (a negative value represents higher expression in Th compared to Treg cells), pFDR: FDR-corrected p-value, Treg: expression level in Treg cells as log_2 value, Th: expression level in Th cells as log_2 value.

Table S5 Probesets regulated by cis-eQTL (LRS >= 18) in Treg but not in Th and expressed at least 2-fold higher in Treg.

Probeset	Gene Symbol	Description	Location (Chr, Mb)	Mean Expr	Max LRS	Max LRS Location (Chr: Mb)
1438091_a_at	*H2afz*	H2A histone family, member Z; distal 3' UTR	Chr3: 137.529816	14.78	62.8	Chr3: 129.527943
1418449_at	*Lad1*	ladinin	Chr1: 137.728962	8.72	60.5	Chr1: 129.064812
1419219_at	*Cyp4f18*	cytochrome P450, family 4, subfamily f, polypeptide 18 (leukotriene-B(4) omega-hydroxylase 2, endoplasmic reticulum, CYP4F3 closed human homolog); exons 9, 10, and 11 (of 12 coding)	Chr8: 74.512987	9.49	58.1	Chr8: 72.331480
1416368_at	*Gsta4*	glutathione S-transferase, alpha 4; last three exons including proximal 3' UTR	Chr9: 78.053722	8.43	54.8	Chr9: 74.382952
1421525_a_at	*Birc1e*	baculoviral IAP repeat-containing 1e; 3' UTR	Chr13: 100.981873	8.70	53.2	Chr13: 99.628243
1427228_at	*Palld*	pallidin actin cytoskeleton organizer; last three exons	Chr8: 63.994000	8.55	41.9	Chr8: 60.181844
1416531_at	*Gsto1*	glutathione S-transferase omega 1 (Alzheimer disease risk factor); last two exons and proximal half of 3' UTR	Chr19: 47.938787	10.71	41.6	Chr19: 47.218628
1435605_at	*Arp3b*	ARP3 actin-related protein 3 homolog B; 3' UTR	Chr5: 25.355920	7.81	39.6	Chr5: 22.716674

1426808_at	*Lgals3*	lectin, galactose binding, soluble 3; last exons and proximal 3' UTR	Chr14: 48.005204	11.45	39.3	Chr14: 47.679976
1430439_at	*2810465F10Rik*	RIKEN cDNA 2810465F10 gene	Chr13: 77.169422	8.07	38.1	Chr13: 75.901603
1426208_x_at	*Plagl1*	pleiomorphic adenoma gene-like 1 (maternally repressed imprinted); distal half of 3' UTR (tandem repeat in UTR)	Chr10: 12.850348	10.98	37.7	Chr10: 12.729006
1435355_at	*AI595938*	expressed sequence AI595938	Chr2: 51.992362	8.39	36.9	Chr2: 43.538349
1455746_at	*Kif13a*	kinesin family member 13A	Chr13: 46.844570	9.31	35.4	Chr13: 52.866221
1451475_at	*Plxnd1*	plexin D1	Chr6: 115.904908	9.39	34.4	Chr6: 115.865551
1423696_a_at	*Psmd6*	proteasome (prosome, macropain) 26S subunit, non-ATPase; last exon and 3' UTR (test Mendelian 14.13)	Chr14: 14.944713	8.61	31.9	Chr14: 8.852063
1421834_at	*Pip5k1b*	phosphatidylinositol-4-phosphate 5-kinase, type 1 beta; proximal to mid 3' UTR	Chr19: 24.369742	8.01	31.7	Chr19: 23.240260
1455771_at	*Bzrap1*	benzodiazapine receptor associated protein 1; mid and distal 3' UTR	Chr11: 87.599094	9.68	31	Chr11: 83.521935
1434600_at	*Tjp2*	tight junction protein 2; mid distal 3' UTR	Chr19: 24.169276	9.72	29.9	Chr19: 23.240260
1422678_at	*Dgat2*	diacylglycerol O-acyltransferase 2; distal 3' UTR	Chr7: 106.302206	8.91	29.6	Chr7: 96.762672
1418392_a_at	*Gbp4*	guanylate nucleotide binding	Chr3: 142.23567	12.91	28.2	Chr3: 142.297853

		protein 4	5			
1422528_a_at	*Zfp36l1*	zinc finger protein 36, C3H type-like 1; mid 3' UTR	Chr12: 81.209730	9.79	27.7	Chr12: 81.069515
1445399_at	*Klrb1d*	killer cell lectin-like receptor subfamily B member 1D	Chr6: 128.763764	7.86	27.2	Chr6: 127.388475
1434277_a_at	*Ypel2*	yippee-like 2; distal 3' UTR	Chr11: 86.749948	11.11	27.1	Chr11: 83.521935
1418910_at	*Bmp7*	bone morphogenetic protein 7	Chr2: 172.695346	8.60	25.9	Chr2: 171.350039
1440779_s_at	*Slc5a9*	solute carrier family 5 (sodium/glucose cotransporter), member 9	Chr4: 111.549922	9.36	25.9	Chr4: 119.150595
1435917_at	*Ociad2*	OCIA domain containing 2; distal 3' UTR	Chr5: 73.713478	8.57	25.7	Chr5: 72.711077
1420421_s_at	*Klrb1d*	killer cell lectin-like receptor subfamily B member 1B; mid-proximal 3' UTR	Chr6: 128.764454	8.08	25.5	Chr6: 127.388475
1420533_at	*Gucy1a3*	guanylate cyclase 1, soluble, alpha 3; last 3 exons and proximal 3' UTR	Chr3: 81.898554	8.13	24.8	Chr3: 77.716616
1428547_at	*Nt5e*	5' nucleotidase, ecto	Chr9: 88.266332	11.55	23.6	Chr9: 86.133451
1434830_at	*Mad*	Max dimerization protein	Chr6: 86.597083	11.33	22	Chr6: 89.298446
1453228_at	*Stx11*	syntaxin 11; distal half of 3' UTR	Chr10: 12.659849	11.26	20.8	Chr10: 12.729006
1434109_at	*Sh3bgrl2*	SH3 domain binding glutamic acid-rich protein like 2; distal 3' UTR (or intron)	Chr9: 83.493312	7.91	19.3	Chr9: 81.753446
1432103_a_at	*Sh3gl3*	SH3-domain GRB2-like 3	Chr7: 89.455550	10.12	18.3	Chr7: 81.491656
1440578_at	*Kif13a*	kinesin family member 13A; intron 2	Chr13: 47.011248	8.33	18.2	Chr13: 52.866221

1429184_at	*Gvin1*	GTPase, very large interferon inducible 1; mid 3' UTR	Chr7: 113.043965	14.36	18.1	Chr7: 111.297535

Probeset: probeset ID of microarray hybridization probe, Symbol: gene symbol in GeneNetwork (note that this may differ from the gene description in MGI), Description: gene description in GeneNetwork (note that this may differ from the gene description in MGI), Location: chromosomal location Chromosome, Megabase of gene, Mean Expr: mean expression level in respective cell type as $\log_2$ value. Max LRS: value of maximum LRS, Max LRS location: position of QTL (Chromosome, Megabase) exhibiting the maximum LRS value.

Table S6 Probesets regulated by cis-eQTL (LRS >= 18) in Th but not in Treg and expressed at least 2-fold higher in Th cells.

Probeset	Gene Symbol	Description	Location (Chr, Mb)	Mean Expr	Max LRS	Max LRS Location (Chr: Mb)
1426780_at	*D14Ertd436e*	DNA segment, Chr 14, ERATO Doi 436, expressed	Chr14: 48.160766	9.40	46.5	Chr14: 47.679976
1458118_at	*5031408O05*	ESTs	Chr2: 91.517597	8.86	41	Chr2: 88.186455
1416700_at	*Rhoe*	ras homolog gene family, member E; distal 3' UTR	Chr2: 50.986172	8.03	38	Chr2: 44.257292
1439736_at	*Itk*	IL2-inducible T-cell kinase; highly expressed intron sequence from EST AK138049	Chr11: 46.191285	10.27	36.4	Chr11: 47.930883
1417069_a_at	*Gmfb*	glia maturation factor, beta; proximal 3' UTR	Chr14: 47.430023	8.72	36.2	Chr14: 47.679976
1435640_x_at	*BE634869*	BE634869 EST sequence; low probeset specificity	Chr11: 86.627587	11.22	35.6	Chr11: 88.522933
1417646_a_at	*Snx5*	sorting nexin 5	Chr2: 144.076822	12.60	34.5	Chr2: 139.549795
1442939_at	*Rif1*	Rap1 interacting factor 1; 5' end of large exon (exon 28)	Chr2: 51.965511	9.13	33.9	Chr2: 44.257292
1426123_a_at	*Rrbp1*	ribosome binding protein 1; 3'UTR of the short mRNA	Chr2: 143.811625	8.59	33.8	Chr2: 139.549795
1438798_at	*4931406P16Rik*	RIKEN cDNA 4931406P16 gene	Chr7: 35.070297	9.40	33.6	Chr7: 31.505577
1447272_s_at	*Atp10a*	ATPase, class V, type 10A	Chr7: 66.084017	9.02	33	Chr7: 63.779523
1448931_at	*F2rl1*	coagulation factor II (thrombin) receptor-like 1	Chr13: 96.281711	10.79	31.8	Chr13: 96.470954
1417152_at	*Btbd14a*	BTB (POZ) domain	Chr2:	8.77	30.5	Chr2:

		containing 14A; proximal 3' UTR	25.914539			25.726695
1429582_at	*Btbd14a*	BTB (POZ) domain containing 14A; distal 3' UTR	Chr2: 25.911274	7.79	30.5	Chr2: 25.726695
1455570_x_ at	*Cnn3*	calponin 3, acidic; distal 3' UTR	Chr3: 121.16092 8	10.18	28.6	Chr3: 119.424849
1433769_at	*Als2cl*	ALS2 C-terminal like	Chr9: 110.80244 0	12.31	28.2	Chr9: 114.377913
1425087_at	*2310003F 16Rik*	RIKEN cDNA 2310003F16 gene	Chr2: 121.28343 0	8.81	27.5	Chr2: 117.118531
1436759_x_ at	*Cnn3*	calponin 3, acidic; distal 3' UTR	Chr3: 121.16102 9	10.05	26.9	Chr3: 119.424849
1436499_at	*Sgms1*	sphingomyelin synthase 1; mid distal 3' UTR	Chr19: 32.197490	12.41	26	Chr19: 32.123499
1454765_at	*Gtf3c3*	general transcription factor IIIC, polypeptide 3; last two exons and proximal 3' UTR	Chr1: 54.454480	9.68	24.6	Chr1: 54.152566
1423059_at	*Ptk2*	PTK2 protein tyrosine kinase 2	Chr15: 73.035903	8.42	24.5	Chr15: 79.746549
1441926_x_ at	*Tmie*	transmembrane inner ear (auditory hair cell maturation)	Chr9: 110.76858 2	9.34	24.5	Chr9: 113.200233
1434427_a_ at	*Rnf157*	Ring finger protein 157; in 3' UTR	Chr11: 116.19772 4	8.40	22.6	Chr11: 114.532621
1439044_at	*Zfp354c*	zinc finger protein 354C	Chr11: 50.624900	7.60	22.5	Chr11: 50.558768
1417153_at	*Btbd14a*	BTB (POZ) domain containing 14A; last 2 exons	Chr2: 25.915618	8.36	22.2	Chr2: 25.726695
1456235_at	*E430004 N04Rik*	RIKEN cDNA E430004N04 gene	Chr10: 28.451052	8.26	22	Chr10: 35.758851
1417377_at	*Igsf4a*	immunoglobulin superfamily, member 4A; mid 3'	Chr9: 47.660213	8.08	22	Chr9: 48.017409

		UTR				
1424426_at	*Mtap*	methylthioadenosine phosphorylase	Chr4: 88.826451	9.46	21.8	Chr4: 87.697880
1452013_at	*Atp10a*	ATPase, class V, type 10A	Chr7: 66.084259	8.54	20.7	Chr7: 63.779523
1431894_at	*Itprip*	inositol 1,4,5-triphosphate receptor interacting protein; intron 1	Chr19: 47.991963	9.57	20	Chr19: 44.265919
1416543_at	*Nfe2l2*	nuclear factor, erythroid derived 2, like 2 (lung damage associated); last exon and 3' UTR	Chr2: 75.513676	11.20	19.6	Chr2: 75.514788
1453244_at	*5830416P10Rik*	RIKEN cDNA 5830416P10 gene	Chr19: 53.536200	9.36	19	Chr19: 53.933992
1439863_at	*Ugcg*	UDP-glucose ceramide glucosyltransferase	Chr4: 59.231205	9.12	18.6	Chr4: 57.626581
1437212_at	*Zfp70*	zinc finger protein cluster member (tentative symbol assignment); mid 3' UTR	Chr7: 30.661718	7.45	18.4	Chr7: 30.566470
1428573_at	*Chn2*	chimerin (chimaerin) 2; distal 3' UTR (long 3' UTR isoform)	Chr6: 54.251276	8.59	18.2	Chr6: 63.773076

See Table S5 for descriptions of columns.

Table S7 Probesets of genes regulated by trans-eQTL (LRS >= 18) in Treg and expressed at least 2-fold higher in Treg than Th cells (DE-2fold genes).

Probeset	Symbol	Description	Location (Chr, Mb)	Mean Expr	Max LRS	Max LRS Location (Chr: Mb)
1436996_x_at	*Lzp-s*	P lysozyme structural and lysozyme; 3' UTRs of both Lzp-s and Lyzs (segmental duplicates)	Chr10: 116.724902	13.23	21.4	Chr8: 98.812565
1424112_at	*Igf2r*	insulin-like growth factor 2 receptor; last exon and proximal 3' UTR	Chr17: 12.876161	12.99	18.7	Chr19: 12.018631
1434690_at	*Lycat*	lysocardiolipin acyltransferase; distal 3' UTR	Chr17: 73.592119	12.02	18.4	Chr6: 17.922616
1428393_at	*Nrn1*	neuritin 1; mid 3' UTR	Chr13: 36.817735	11.63	18.3	Chr4: 129.249414
1419759_at	*Abcb1a*	ATP-binding cassette, sub-family B (MDR/TAP), member 1A (P-glycoprotein); distal 3' UTR	Chr5: 8.748049	11.60	19.3	Chr16: 34.308445
1425584_x_at	*BC010605*	BC010605, non-coding; unknown, possible alignment error	Chr6: 68.193103	11.18	39.7	Chr16: 39.118133
1439426_x_at	*Lyzs*	lysozyme and P lysozyme structural; distal 3' UTRs of Lzp-s and 3' UTR of Lyzs (segmental duplicates)	Chr10: 116.687814	11.08	22.2	Chr8: 98.812565
1423547_at	*Lyzs*	lysozyme; 5' and 3' UTR	Chr10: 116.714747	10.68	20.4	Chr8: 96.926410
1449232_at	*Gata1*	GATA binding protein 1	ChrX: 7.536411	10.35	18.2	Chr19: 53.933992
1416148_at	*Laptm4b*	lysosomal-associated protein transmembrane 4B;	Chr15: 34.213513	9.88	18.7	Chr2: 102.941478

		mid 3' UTR				
1428650_at	*Tns1*	tensin 1; distal 3' UTR	Chr1: 73.956877	9.52	18.2	Chr5: 104.572763
1441930_x_at	*Vat1*	vesicle amine transport protein 1 homolog (T californica)	Chr11: 101.320123	8.94	18.5	Chr18: 77.065227
1439831_at	*3200002M19Rik*	RIKEN cDNA 3200002M19 gene	Chr18: 60.407662	8.91	21.9	Chr5: 100.230580
1423403_at	*Mapkbp1*	mitogen activated protein kinase binding protein 1	Chr2: 119.852734	8.81	18.2	Chr7: 122.555416
1441643_at	*March3*	membrane-associated ring finger (C3HC4) 3	Chr18: 57.081968	8.45	18.4	Chr16: 32.679562
1449498_at	*Marco*	macrophage receptor with collagenous structure	Chr1: 122.371217	8.35	19.8	Chr8: 96.926410
1419117_at	*Slc22a2*	solute carrier family 22 (organic cation transporter), member 2	Chr17: 12.812677	8.35	18.8	Chr19: 22.531308
1432057_a_at	*Prdm5*	PR domain containing 5; last 3 exons of long form	Chr6: 65.885964	8.24	19.8	Chr5: 14.324112
1448690_at	*Kcnk1*	potassium channel, subfamily K, member 1	Chr8: 128.554041	8.21	18.6	Chr7: 30.566470
1442050_at	*Znf608*	zinc finger protein 608; last exons and proximal 3' UTR	Chr18: 55.048400	7.79	20.2	Chr2: 17.962147
1455034_at	*Nr4a2*	nuclear receptor subfamily 4, group A, member 2; distal 3' UTR	Chr2: 56.959322	7.79	19	Chr6: 86.579759
1455978_a_at	*Matn2*	matrilin 2; distal 3' UTR	Chr15: 34.365882	7.48	32	Chr17: 10.586983
1451826_at	*Cabp5*	calcium binding protein 5 (retina specific); distal half of 3' UTR	Chr7: 13.993739	7.39	18.2	Chr1: 128.625710

1419442_at	*Matn2*	matrilin 2	Chr15: 34.362461	7.27	23.1	Chr17: 10.586983
1421430_at	*Rad51l1*	RAD51-like 1 (S. cerevisiae)	Chr12: 80.868056	7.16	18	Chr5: 56.064119

See Table S5 for descriptions of columns.

Table S8 Probesets regulated by trans-eQTL (LRS >= 18) in Th and expressed at least 2-fold higher in Th cells.

Probeset	Symbol	Description	Location (Chr, Mb)	Mean Expr	Max LRS	Max LRS Location (Chr: Mb)
1419481_at	*Sell*	selectin, lymphocyte	Chr1: 166.009816	13.01	18.4	Chr18: 82.409996
1455695_at	*St8sia1*	ST8 alpha-N-acetyl-neuraminide alpha-2,8-sialyltransferase 1; distal 3' UTR	Chr6: 142.770075	12.43	20.8	Chr7: 62.999954
1436182_at	*Satb1*	special AT-rich sequence binding protein 1; putative far 3' UTR	Chr17: 51.875670	12.29	23.6	Chr7: 63.779523
1453568_at	*Eeda*	early epithelial differentiation-associated; last three exons	Chr2: 59.332531	10.90	22.4	Chr6: 135.272116
1422478_a_at	*Acas2*	acetyl-Coenzyme A synthetase 2 (ADP forming); distal 3' UTR	Chr2: 155.388318	10.81	21.1	Chr18: 84.295655
1435536_at	*1700027M01Rik*	RIKEN cDNA 1110056G13 gene	Chr4: 141.233560	10.61	19.6	Chr5: 33.660747
1451122_at	*Idi1*	isopentenyl-diphosphate delta isomerase (peroxisomal enzyme, cholesterol synthesis); distal 3' UTR	Chr13: 8.891078	10.50	20.1	Chr17: 43.961584
1421992_a_at	*Igfbp4*	insulin-like growth factor binding protein 4, differentially expressed in B16F10 2; mid 3' UTR	Chr11: 98.913030	10.07	19.4	Chr7: 127.836779
1451862_a_at	*Prf1*	perforin 1 (pore forming protein)	Chr10: 60.766209	9.79	18.8	Chr1: 87.687978

1449972_s_at	*BC018101*	cDNA sequence BC018101; 3' UTR (transQTL on Chr 14 in BXD hippocampus data)	Chr17: 17.226126	9.72	29.8	Chr14: 8.852063
1437375_at	*Rfx3*	regulatory factor X, 3 (dentate gyrus expression signature, influences HLA class II expression); possible far 3' UTR of Rfx3	Chr19: 27.836277	9.64	21.9	Chr7: 73.316123
1428737_s_at	*9130427A09Rik*	RIKEN cDNA 9130427A09 gene	Chr18: 56.651549	9.51	24.1	Chr9: 98.479729
1420401_a_at	*Ramp3*	receptor (calcitonin) activity modifying protein 3; proximal to mid 3' UTR	Chr11: 6.576916	9.44	18.7	Chr5: 113.832344
1449903_at	*Crtam*	cytotoxic and regulatory T cell molecule	Chr9: 40.780888	9.40	18.2	Chr13: 68.711507
1434402_at	*Samd8*	sterile alpha motif domain containing 8; distal 3' UTR	Chr14: 22.617386	9.29	20.1	Chr7: 62.999954
1446570_at	*Maml2*	mastermind-like 2; intron (from AK084324)	Chr9: 13.457782	9.22	22.3	Chr1: 79.802768
1418843_at	*Slc30a4*	solute carrier family 30 (zinc transporter), member 4; distal 3' UTR	Chr2: 122.507068	9.18	18.1	Chr17: 44.224864
1457390_at	*Prpf3*	PRP3 pre-mRNA processing factor 3 (retinitis pigmentosa); first intron or promoter region	Chr3: 95.657836	9.12	18.3	Chr7: 90.186486
1456822_at	*Rad23b*	RAD23b homolog (S. cerevisiae)	Chr4: 55.380895	8.88	19.8	Chr10: 103.024850
1425713_a_at	*Rnf146*	ring finger protein 146; last exon	Chr10: 29.066989	8.85	20	Chr7: 19.794682

1435554_at	*Tmcc3*	transmembrane and coiled coil domains 3	Chr10: 94.053203	8.78	23.1	Chr4: 66.843058
1448485_at	*Ggt1*	gamma-glutamyltransferase 1; last four exons and 3' UTR	Chr10: 75.048010	8.76	18.2	Chr9: 97.259752
1446886_at	*Usp3*	ubiquitin specific protease 3	Chr9: 66.413157	8.74	18.8	Chr19: 48.972881
1442750_at	*B3galnt2*	UDP-GalNAc:betaGlcNAc beta 1,3-galactosaminyltransferase, polypeptide 2	Chr13: 14.064363	8.68	20.2	Chr2: 74.927702
1437822_at	*Yme1l1*	YME1-like 1 (S. cerevisiae)	Chr2: 23.032027	8.41	21.3	Chr1: 79.802768
1419585_at	*Rp2*	retinitis pigmentosa 2 (X-linked recessive); last two exons and proximal 3' UTR	ChrX: 19.974379	7.77	20.3	Chr16: 32.679562
1446412_at	*Wwox*	WW domain-containing oxidoreductase	Chr8: 117.339587	7.68	19.6	ChrX: 68.694214
1421014_a_at	*Clybl*	citrate lyase beta like; exons 5 and 6	Chr14: 122.778423	7.63	35.4	Chr4: 152.699719
1459923_at	*5830435K17Rik*	Mus musculus transcribed sequences	Chr16: 32.186774	7.38	19.9	Chr4: 155.495856
1424567_at	*Tspan2*	tetraspan 2; mid 3' UTR	Chr3: 102.574565	7.17	21.4	Chr16: 31.615881

See Table S5 for descriptions of columns.

Table S9 Probesets of genes differentially expressed in Treg cells with a known Treg function and a high expression signal in Tregs (expression signal > 8) and LRS >=14.

**Probeset**	**Symbol**	**Description**	**Location (Chr, Mb)**	**Mean Expr**	**Max LRS**	**Max LRS Location (Chr: Mb)**
1448951_at	*Tnfrsf1b*	tumor necrosis factor receptor superfamily, member 1b; last 2 exons	Chr4: 144.805686	8.65	18.2	Chr3: 26.093916
1418084_at	*Nrp1*	neuropilin 1; 3' UTR	Chr8: 131.026840	12.77	16.4	Chr2: 77.938377
1418099_at	*Tnfrsf1b*	tumor necrosis factor receptor superfamily, member 1b	Chr4: 144.803453	11.50	15.3	Chr18: 76.899558
1460700_at	*Stat3*	signal transducer and activator of transcription 3; distal 3' UTR	Chr11: 100.748537	11.45	14.5	Chr7: 30.566470
1448943_at	*Nrp1*	neuropilin 1; distal 3' UTR	Chr8: 131.028801	13.19	14.3	ChrX: 68.694214
1457342_at	*Ikzf4*	IKAROS family zinc finger 4 (Eos); distal 3' UTR	Chr10: 128.067934	11.90	14.1	Chr7: 126.800203
1457198_at	*Nrp1*	neuropilin 1; intron 2 or possible short form 3' UTR	Chr8: 130.888760	12.03	14	Chr9: 62.226499

See Table S5 for descriptions of columns.

Table S10: Intervals selected for further QTL analysis

Gene	QTL in Treg	QTL in Th	Treg QTL chr	Treg QTL region	Th QTL chr	Th QTL region
Nrp1	trans		2	75-80		
Tnfrs1b	trans		3	20-30		
F2rl1	trans		4	28-35		
Klrb1f	trans		X	90-110		
Clta4		trans			17	25-45
Stx11		trans			17	25-45
Laptm4b	trans	trans	2	100-130	7	65-80
Lycat	trans	trans	6	10-25	17	25-45
Prpf3	trans	trans	7	75-90	7	75-90

Table S11 Probesets of genes located in the QTL interval on chromosome 4 regulating the expression of F2rl1 and exhibiting an expression signal larger than 8.

Probeset	Symbol	Description	Location (Chr, Mb)	Mean Expr	Max LRS	Max LRS Location (Chr: Mb)
1433270_at	*9530004 M16Rik*	RIKEN cDNA 9530004M16 gene	Chr4: 32.095144	8.37	11.8	Chr2: 80.455843
1425795_a_at	*Map3k7*	mitogen activated protein kinase kinase kinase 7; proximal half of 3' UTR	Chr4: 32.106640	8.73	9.5	Chr13: 35.965560
1419988_at	*Map3k7*	mitogen activated protein kinase kinase kinase 7; mid distal 3' UTR	Chr4: 32.107395	8.98	12.9	Chr6: 16.932017
1455441_at	*Map3k7*	mitogen activated protein kinase kinase kinase 7; far 3' UTR	Chr4: 32.109924	9.07	48.6	Chr4: 31.651725
1446929_at	*Bach2*	BTB and CNC homology 2	Chr4: 32.333020	10.06	13	Chr15: 56.992039
1421598_at	*Bach2*	BTB and CNC homology 2	Chr4: 32.475137	8.04	9.5	Chr2: 30.296486
1442605_at	*Bach2*	BTB and CNC homology 2	Chr4: 32.541406	8.73	8.8	Chr6: 149.016661
1440304_at	*Bach2*	BTB and CNC homology 2	Chr4: 32.543500	10.93	7.5	Chr15: 56.992039
1439468_at	*Bach2*	BTB and CNC homology 2	Chr4: 32.594530	10.17	8.6	Chr9: 69.810185
1443263_at	*Bach2*	BTB and CNC homology 2	Chr4: 32.627277	10.14	15.2	Chr15: 56.992039
1437667_a_at	*Bach2*	BTB and CNC homology 2	Chr4: 32.667776	11.56	17.7	Chr10: 19.804439
1445460_at	*Bach2*	ESTs	Chr4: 32.670286	8.17	15	Chr3: 144.425237
1436515_at	*E030004 N02Rik*	RIKEN cDNA E030004N02 gene	Chr4: 32.672630	10.95	15.8	Chr12: 109.609890
1449217_at	*Casp8ap2*	caspase 8 associated protein 2	Chr4: 32.739897	11.27	11.1	Chr12: 105.632190

1417675_a_at	*Mdn1*	midasin homolog; distal half oof 3' UTR	Chr4: 32.861595	10.77	8.3	Chr9: 105.676711
1427935_at	*2610208E05Rik*	RIKEN cDNA 2610208E05 gene	Chr4: 32.887476	9.95	13.2	Chr7: 94.381202
1427934_at	*2610208E05Rik*	complex 1 protein (LYR family); mid distal 3' UTR	Chr4: 32.888797	9.84	9.4	Chr2: 79.183599
1437217_at	*Ankrd6*	ankyrin repeat domain 6; distal 3' UTR	Chr4: 32.891171	8.54	10.1	Chr18: 28.455185
1434909_at	*C030003H22Rik*	RIKEN cDNA C030003H22 gene	Chr4: 33.108578	10.46	10.5	Chr2: 44.257292
1417723_at	*Ube2j1*	ubiquitin-conjugating enzyme E2, J1; distal 3' UTR	Chr4: 33.138805	12.02	10.2	Chr6: 50.804367
1433668_at	*Pnrc1*	proline rich 2	Chr4: 33.332548	13.06	11.6	Chr3: 26.093916
1425844_a_at	*Rngtt*	RNA guanylyltransferase and 5'-phosphatase	Chr4: 33.530578	9.63	13	Chr6: 149.016661
1428237_at	*Ccdc111*	coiled-coil domain containing 111; distal 3' UTR	Chr4: 34.513582	12.50	18.9	Chr4: 32.187427
1416117_at	*Orc3l*	origin recognition complex, subunit 3-like (S. cerevisiae)	Chr4: 34.516538	8.05	11	Chr6: 95.023509
1416116_at	*Orc3l*	origin recognition complex, subunit 3-like (S. cerevisiae)	Chr4: 34.517296	8.24	9.9	Chr14: 47.679976
1416115_at	*Orc3l*	origin recognition complex, subunit 3-like (S. cerevisiae)	Chr4: 34.519713	10.54	11.3	Chr4: 32.187427
1435719_at	*AI448984*	adult male hypothalamus cDNA, RIKEN full-length enriched library, clone:A230020J12 product:origin of replication 3 homolog (S. cerevisiae), full	Chr4: 34.527438	8.16	18	Chr4: 33.109559

		insert sequence.				
1424209_at	*Rarsl*	arginyl-tRNA synthetase-like; exons 15, 16, 17, 18 19, 20	Chr4: 34.603314	10.60	9.1	Chr5: 59.037549
1417538_at	*Slc35a1*	solute carrier family 35 (CMP-sialic acid transporter), member 1	Chr4: 34.610664	10.54	16.8	Chr2: 79.183599
1417539_at	*Slc35a1*	solute carrier family 35 (CMP-sialic acid transporter), member 1; exons 6 and 7	Chr4: 34.616289	8.54	11.8	Chr2: 17.962147
1430292_a_at	*1810030N24Rik*	RIKEN cDNA 1810030N24 gene	Chr4: 34.716185	9.12	32.6	Chr4: 34.041018
1436308_at	*Zfp292*	zinc finger protein 292; mid distal 3' UTR	Chr4: 34.750806	10.43	17.9	Chr7: 81.491656
1419575_s_at	*Zfp292*	zinc finger protein 292; mid 3' UTR	Chr4: 34.751323	10.94	15	Chr4: 33.109559
1419574_at	*Zfp292*	zinc finger protein 292; 3' UTR	Chr4: 34.751571	9.74	10.8	Chr4: 45.985763
1449515_at	*Zfp292*	zinc finger protein 292; exons and proximal 3' UTR	Chr4: 34.752137	10.72	15.5	Chr4: 34.041018
1430216_at	*5730450D02Rik*	RIKEN cDNA 5730450D02 gene	Chr4: 34.758916	8.82	16.2	Chr16: 32.679562
1457842_at	*LOC229512*	ESTs	Chr4: 34.774664	10.67	9.3	ChrX: 150.143066

See Table S5 for descriptions of columns.

Table S12 Probesets of genes located in the QTL interval on chromosome 2 (autoimmunity to DNA), expressed in Treg (signal > 8) and exhibiting a cis-eQTL of LRS >=15.

Record	Symbol	Description	Location (Chr, Mb)	Mean Expr	Max LRS	Max LRS Location (Chr: Mb)
1451492_at	*Sla2*	Src-like-adaptor 2	Chr2: 156.699913	9.68	19.9	Chr2: 156.689629
1417663_a_at	*Ndrg3*	N-myc downstream regulated gene 3; distal 3' UTR	Chr2: 156.753126	9.19	29.8	Chr2: 156.689629
1450918_s_at	*Src*	Rous sarcoma oncogene; distal 3' UTR	Chr2: 157.297175	8.20	16.8	Chr2: 157.820958
1455080_at	*Ppp1r16b*	protein phosphatase 1, regulatory (inhibitor) subunit 16B; distal 3' UTR	Chr2: 158.591508	12.09	33	Chr2: 157.820958
1438495_at	*Top1*	topoisomerase (DNA) I; intron	Chr2: 160.475245	8.99	16	Chr2: 165.608227
1417976_at	*Ada*	adenosine deaminase	Chr2: 163.552611	8.75	24	Chr2: 164.559755
1448377_at	*Slpi*	secretory leukocyte protease inhibitor; last two exons and 3' UTR (proximal and mid)	Chr2: 164.180612	8.78	19.7	Chr2: 164.559755
1438206_a_at	*2610042O14Rik*	RIKEN cDNA 2610042O14 gene	Chr2: 164.289987	11.61	28.7	Chr2: 164.559755
1450057_at	*2610042O14Rik*	RIKEN cDNA 2610042O14 gene	Chr2: 164.290426	9.69	17.6	Chr2: 164.559755
1453039_at	*Znf335*	RIKEN cDNA 1810045J01 gene	Chr2: 164.717422	9.57	18.5	Chr2: 164.559755
1442408_at	*Sulf2*	sulfatase 2; distal 3' UTR (antisense in distal 3' UTR of	Chr2: 165.89814	12.43	19.2	Chr2: 165.608227

		Ncoa3)	3			
1448027_at	*Ncoa3*	nuclear receptor coactivator 3	Chr2: 165.898173	10.79	21.1	Chr2: 169.778380
1443387_at	*BB045044*	ESTs	Chr2: 166.507344	8.78	27.7	Chr2: 168.349119
1435347_at	*Stau1*	staufen (RNA binding protein) 1; far 3' UTR	Chr2: 166.773161	9.61	16.8	Chr2: 165.608227
1427604_a_at	*Atp9a*	ATPase, class 2, type 9A; last three exons	Chr2: 168.463054	8.52	20.2	Chr2: 165.608227
1435479_at	*Bmp7*	bone morphogenetic protein 7	Chr2: 172.693765	8.44	24.3	Chr2: 169.778380
1422706_at	*Tmepai*	transmembrane, prostate androgen induced RNA; proximal 3' UTR	Chr2: 173.052359	10.72	22.9	Chr2: 168.349119
1422705_at	*Tmepai*	transmembrane, prostate androgen induced RNA	Chr2: 173.053526	8.59	23.9	Chr2: 168.349119
1438783_at	*Tmepai*	transmembrane, prostate androgen induced RNA	Chr2: 173.098599	8.62	27.2	Chr2: 168.349119

See Table S5 for descriptions of columns.

References

1. Fontenot JD, Gavin MA, Rudensky AY: **Foxp3 programs the development and function of CD4+CD25+ regulatory T cells**. *Nat Immunol* 2003, **4**(4):330-336.
2. Sakaguchi S, Sakaguchi N, Asano M, Itoh M, Toda M: **Immunologic self-tolerance maintained by activated T cells expressing IL-2 receptor alpha-chains (CD25). Breakdown of a single mechanism of self-tolerance causes various autoimmune diseases**. *J Immunol* 1995, **155**(3):1151-1164.
3. Hansen W, Loser K, Westendorf AM, Bruder D, Pfoertner S, Siewert C, Huehn J, Beissert S, Buer J: **G protein-coupled receptor 83 overexpression in naive CD4+CD25- T cells leads to the induction of Foxp3+ regulatory T cells in vivo**. *J Immunol* 2006, **177**(1):209-215.

4. Bruder D, Probst-Kepper M, Westendorf AM, Geffers R, Beissert S, Loser K, von Boehmer H, Buer J, Hansen W: **Neuropilin-1: a surface marker of regulatory T cells**. *Eur J Immunol* 2004, **34**(3):623-630.
5. Pan F, Yu H, Dang EV, Barbi J, Pan X, Grosso JF, Jinasena D, Sharma SM, McCadden EM, Getnet D *et al*: **Eos mediates Foxp3-dependent gene silencing in CD4+ regulatory T cells**. *Science* 2009, **325**(5944):1142-1146.
6. Shimizu J, Yamazaki S, Takahashi T, Ishida Y, Sakaguchi S: **Stimulation of CD25(+)CD4(+) regulatory T cells through GITR breaks immunological self-tolerance**. *Nat Immunol* 2002, **3**(2):135-142.
7. Collison LW, Workman CJ, Kuo TT, Boyd K, Wang Y, Vignali KM, Cross R, Sehy D, Blumberg RS, Vignali DA: **The inhibitory cytokine IL-35 contributes to regulatory T-cell function**. *Nature* 2007, **450**(7169):566-569.
8. Wing K, Onishi Y, Prieto-Martin P, Yamaguchi T, Miyara M, Fehervari Z, Nomura T, Sakaguchi S: **CTLA-4 control over Foxp3+ regulatory T cell function**. *Science* 2008, **322**(5899):271-275.
9. Liu G, Burns S, Huang G, Boyd K, Proia RL, Flavell RA, Chi H: **The receptor S1P1 overrides regulatory T cell-mediated immune suppression through Akt-mTOR**. *Nat Immunol* 2009, **10**(7):769-777.
10. Cao X, Cai SF, Fehniger TA, Song J, Collins LI, Piwnica-Worms DR, Ley TJ: **Granzyme B and perforin are important for regulatory T cell-mediated suppression of tumor clearance**. *Immunity* 2007, **27**(4):635-646.
11. Garin MI, Chu CC, Golshayan D, Cernuda-Morollon E, Wait R, Lechler RI: **Galectin-1: a key effector of regulation mediated by CD4+CD25+ T cells**. *Blood* 2007, **109**(5):2058-2065.
12. Lehmann J, Huehn J, de la Rosa M, Maszyna F, Kretschmer U, Krenn V, Brunner M, Scheffold A, Hamann A: **Expression of the integrin alpha Ebeta 7 identifies unique subsets of CD25+ as well as CD25- regulatory T cells**. *Proc Natl Acad Sci U S A* 2002, **99**(20):13031-13036.
13. Huang CT, Workman CJ, Flies D, Pan X, Marson AL, Zhou G, Hipkiss EL, Ravi S, Kowalski J, Levitsky HI *et al*: **Role of LAG-3 in regulatory T cells**. *Immunity* 2004, **21**(4):503-513.
14. Borsellino G, Kleinewietfeld M, Di Mitri D, Sternjak A, Diamantini A, Giometto R, Hopner S, Centonze D, Bernardi G, Dell'Acqua ML *et al*: **Expression of ectonucleotidase CD39 by Foxp3+ Treg cells: hydrolysis of extracellular ATP and immune suppression**. *Blood* 2007, **110**(4):1225-1232.
15. Deaglio S, Dwyer KM, Gao W, Friedman D, Usheva A, Erat A, Chen JF, Enjyoji K, Linden J, Oukka M *et al*: **Adenosine generation catalyzed by CD39 and CD73 expressed on regulatory T cells mediates immune suppression**. *J Exp Med* 2007, **204**(6):1257-1265.
16. Chaudhry A, Rudra D, Treuting P, Samstein RM, Liang Y, Kas A, Rudensky AY: **CD4+ regulatory T cells control TH17 responses in a Stat3-dependent manner**. *Science* 2009, **326**(5955):986-991.
17. Zheng Y, Chaudhry A, Kas A, deRoos P, Kim JM, Chu TT, Corcoran L, Treuting P, Klein U, Rudensky AY: **Regulatory T-cell suppressor program co-opts transcription factor IRF4 to control T(H)2 responses**. *Nature* 2009, **458**(7236):351-356.
18. Cretney E, Xin A, Shi W, Minnich M, Masson F, Miasari M, Belz GT, Smyth GK, Busslinger M, Nutt SL *et al*: **The transcription factors Blimp-1 and IRF4 jointly control the differentiation and function of effector regulatory T cells**. *Nat Immunol* 2011, **12**(4):304-311.
19. Chen X, Subleski JJ, Kopf H, Howard OM, Mannel DN, Oppenheim JJ: **Cutting edge: expression of TNFR2 defines a maximally suppressive subset of mouse CD4+CD25+FoxP3+ T regulatory cells: applicability to tumor-infiltrating T regulatory cells**. *J Immunol* 2008, **180**(10):6467-6471.

20. Sugai M, Aoki K, Osato M, Nambu Y, Ito K, Taketo MM, Shimizu A: **Runx3 is required for full activation of regulatory T cells to prevent colitis-associated tumor formation**. *J Immunol* 2011, **186**(11):6515-6520.
21. Kuczma M, Lee JR, Kraj P: **Connexin 43 signaling enhances the generation of foxp3+ regulatory T cells**. *J Immunol* 2011, **187**(1):248-257.
22. Patterson SJ, Han JM, Garcia R, Assi K, Gao T, O'Neill A, Newton AC, Levings MK: **Cutting Edge: PHLPP Regulates the Development, Function, and Molecular Signaling Pathways of Regulatory T Cells**. *J Immunol* 2011, **186**(10):5533-5537.
23. Probst-Kepper M, Geffers R, Kroger A, Viegas N, Erck C, Hecht HJ, Lunsdorf H, Roubin R, Moharregh-Khiabani D, Wagner K *et al*: **GARP: a key receptor controlling FOXP3 in human regulatory T cells**. *J Cell Mol Med* 2009, **13**(9B):3343-3357.
24. Wang R, Kozhaya L, Mercer F, Khaitan A, Fujii H, Unutmaz D: **Expression of GARP selectively identifies activated human FOXP3+ regulatory T cells**. *Proc Natl Acad Sci U S A* 2009, **106**(32):13439-13444.
25. Tran DQ, Andersson J, Hardwick D, Bebris L, Illei GG, Shevach EM: **Selective expression of latency-associated peptide (LAP) and IL-1 receptor type I/II (CD121a/CD121b) on activated human FOXP3+ regulatory T cells allows for their purification from expansion cultures**. *Blood* 2009, **113**(21):5125-5133.
26. Kubach J, Lutter P, Bopp T, Stoll S, Becker C, Huter E, Richter C, Weingarten P, Warger T, Knop J *et al*: **Human CD4+CD25+ regulatory T cells: proteome analysis identifies galectin-10 as a novel marker essential for their anergy and suppressive function**. *Blood* 2007, **110**(5):1550-1558.
27. Tone Y, Furuuchi K, Kojima Y, Tykocinski ML, Greene MI, Tone M: **Smad3 and NFAT cooperate to induce Foxp3 expression through its enhancer**. *Nat Immunol* 2008, **9**(2):194-202.
