# Supplementary material for: Expression QTL mapping in regulatory and helper T cells from the BXD family of strains reveals novel cell-specific genes, gene-gene interactions and candidate genes for auto-immune disease
Source: BMC Genomics. 2011 Dec 19;12:610. doi: 10.1186/1471-2164-12-610 (PMC3277499; doi:10.1186/1471-2164-12-610)
Supplement: Additional file 4 — Figure S1. Genome-wide graph of cis- and trans-eQTLs in Treg and Th cells. The file shows the genome-wide mapping graphs for Treg cells and the same graph for Th cells overlaid in grey. Figure S2. Genome-wide graph of cis- and trans-eQTLs in Th and Treg cells. The file shows the genome-wide mapping graphs for Th cells and the same graph for Treg cells overlaid in grey. Figure S3, Genome wide eQTL mapping of Lycat transcript in Treg and Th cells. The file contains a graph showing the result of a genome-wide mapping of eQTLs for the Lycat transcript in Treg and Th cells. Figure S4, Genome wide eQTL mapping of Prpf3 transcript in Treg and Th cells. The file contains a graph showing the result of a genome-wide mapping of eQTLs for the Prpf3 transcript in Treg and Th cells. Figure S5, Genome wide eQTL mapping of Nrp1 transcript in Treg and Th cells. The file contains a graph showing the result of a genome-wide mapping of eQTLs for the Nrp1 transcript in Treg and Th cells. Figure S6, Genome wide eQTL mapping of F2rl1 transcript in Treg and Th cells. The file contains a graph showing the result of a genome-wide mapping of eQTLs for the F2rl1 transcript in Treg and Th cells. Figure S7, Genome wide eQTL mapping of Ctla4 transcript in Treg and Th cells. The file contains a graph showing the result of a genome-wide mapping of eQTLs for the Ctla4 transcript in Treg and Th cells. [file 1471-2164-12-610-S4.PDF]

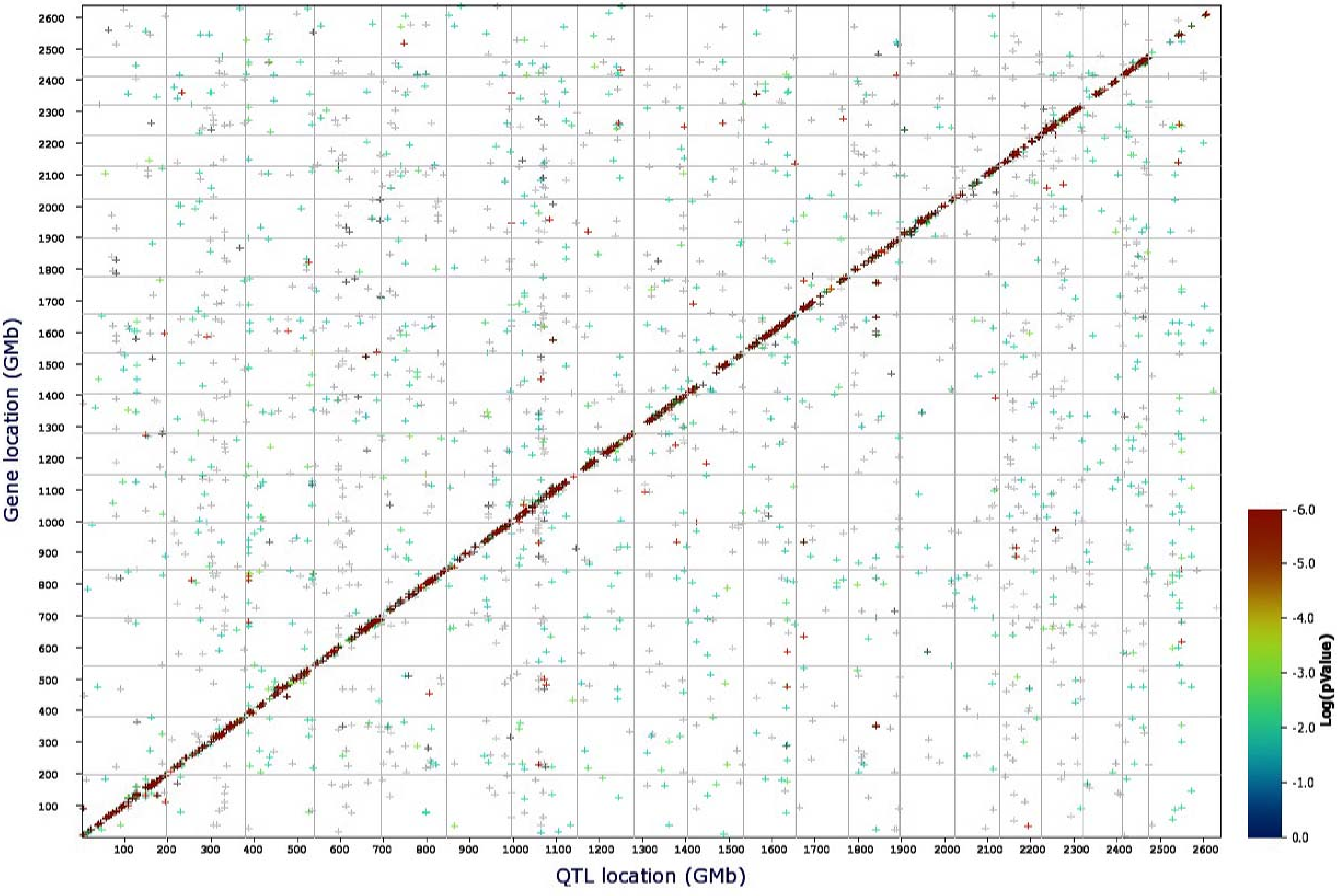

Gene location (GMb)
QTL location (GMb)
Log(pValue)
-6.0
-5.0
-4.0
-3.0
-2.0
-1.0
0.0
100
200
300
400
500
600
700
800
900
1000
1100
1200
1300
1400
1500
1600
1700
1800
1900
2000
2100
2200
2300
2400
2500
2600

Figure S1 Genome-wide graph of cis- and trans-eQTLs in Treg and Th cells.

The positions of the eQTLs are plotted against the locations of the corresponding transcript along the genome. Cis-regulated genes are located along the diagonal, all other dots represent trans-regulated genes. The significance level of each QTL is indicated by the color. FDR = 0.3. Positions for Treg cells are represented in color, signals for Th cells are overlaid in black and white.

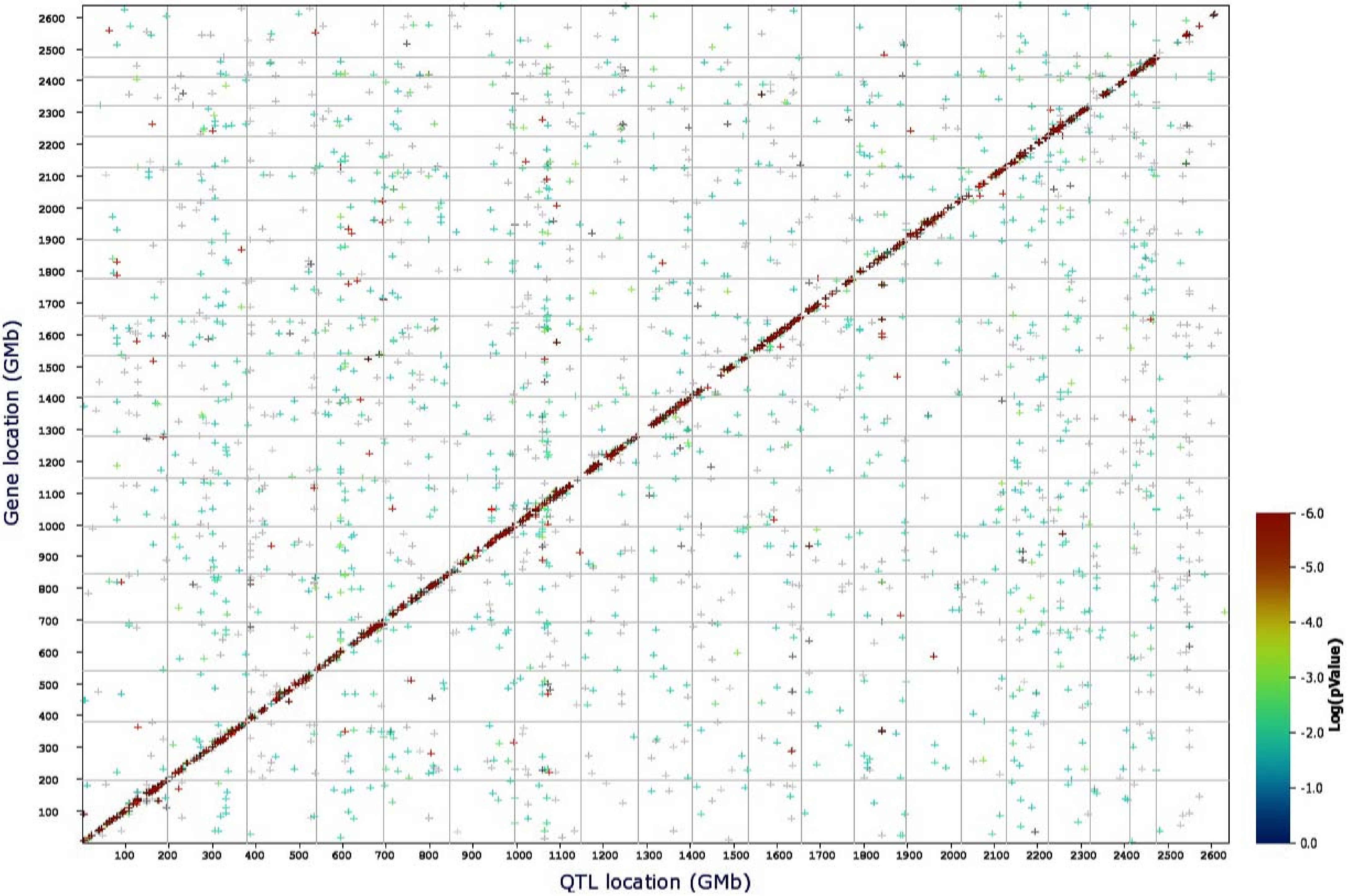

Gene location (GMb)
QTL location (GMb)
Log(pValue)
-6.0
-5.0
-4.0
-3.0
-2.0
-1.0
0.0

Figure S2 Genome-wide graph of cis- and trans-eQTLs in Th and Treg cells.

The positions of the eQTLs are plotted against the locations of the corresponding transcript along the genome. Cis-regulated genes are located along the diagonal, all other dots represent trans-regulated genes. The significance level of each QTL is indicated by the color. FDR = 0.3. Positions for Th cells are represented in color, signals for Treg cells are overlaid in black and white.

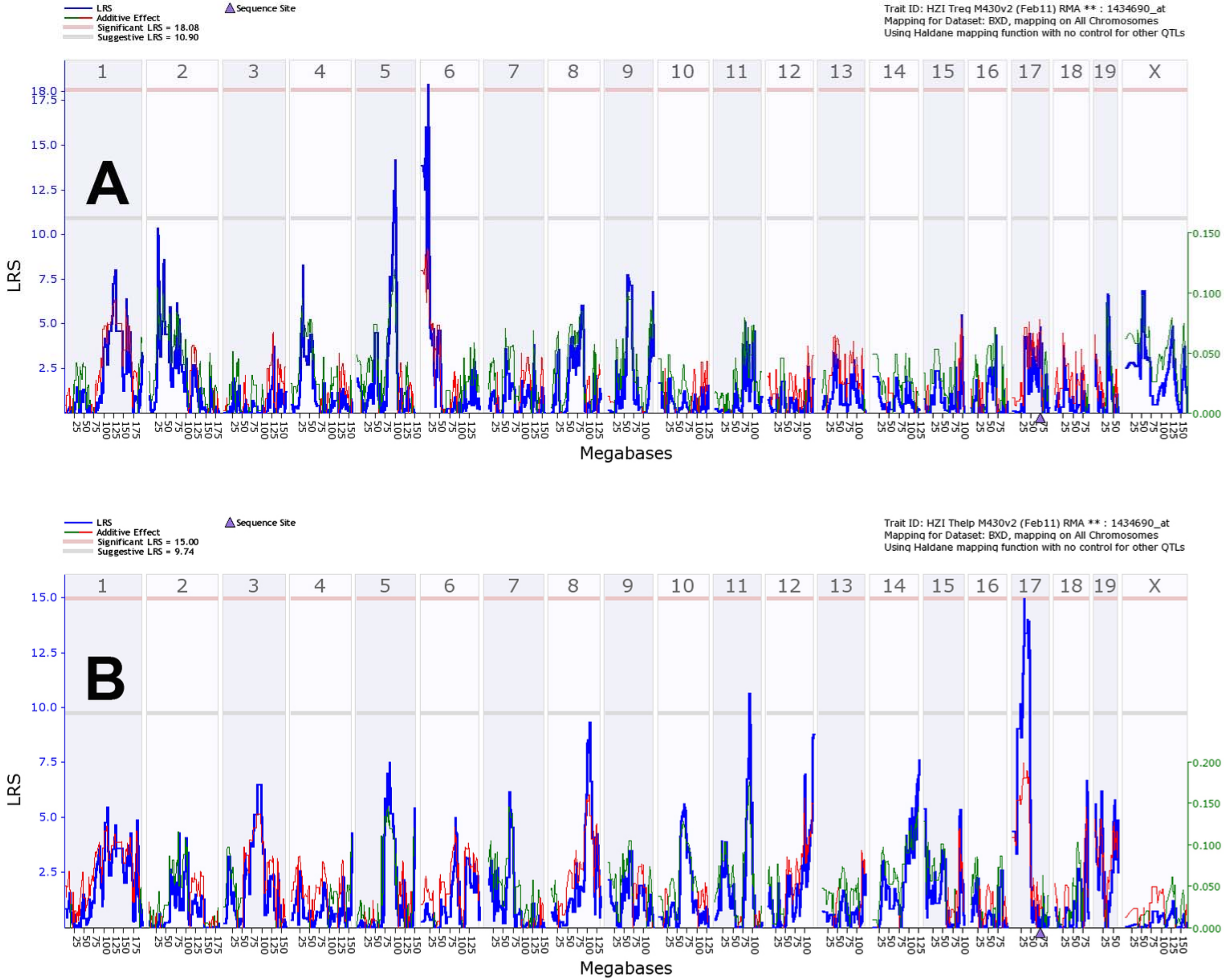

LRS
Additive Effect
Significant LRS = 18.08
Suggestive LRS = 10.90
Sequence Site
Trait ID: HZI Treg M430v2 (Feb11) RMA ** : 1434690_at
Mapping for Dataset: BXD, mapping on All Chromosomes
Using Haldane mapping function with no control for other QTLs
A
LRS
Megabases
LRS
Additive Effect
Significant LRS = 15.00
Suggestive LRS = 9.74
Sequence Site
Trait ID: HZI Thelp M430v2 (Feb11) RMA ** : 1434690_at
Mapping for Dataset: BXD, mapping on All Chromosomes
Using Haldane mapping function with no control for other QTLs
B
LRS
Megabases

Figure S3 Genome wide eQTL mapping of Lycat transcript in Treg and Th cells.

(A) eQTL map for *Lycat* (probeset 1434690_at in Tregs and (B) in Th, respectively. The numbers at the top are chromosomes, and positions at the bottom are given in megabases along the chromosome. The blue line represents the significance level of the QTL expressed as LRS score (likelihood ratio statistic). A positive additive coefficient (green line) indicates that DBA/2J alleles increased trait values. A negative additive coefficient (red line) indicates that C57BL/6J alleles increased trait values. The two horizontal lines mark the genome-wide significance levels at $p<0.05$ (red line) and $p<0.37$ (gray line). A blue triangle marks the position of the gene.

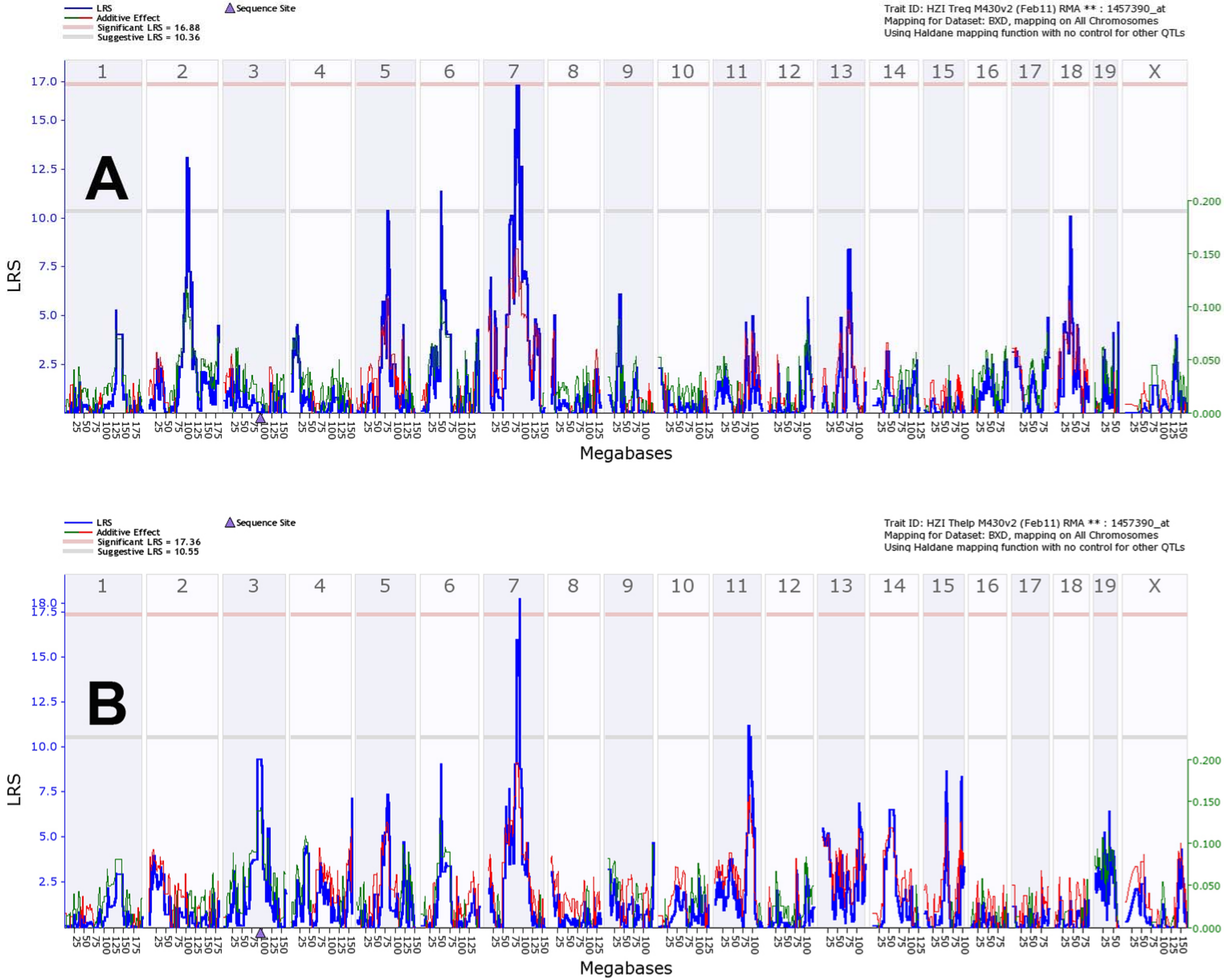
LRS
Additive Effect
Significant LRS = 16.88
Suggestive LRS = 10.36
Sequence Site
Trait ID: HZI Treg M430v2 (Feb11) RMA ** : 1457390_at
Mapping for Dataset: BXD, mapping on All Chromosomes
Using Haldane mapping function with no control for other QTLs
A
LRS
Megabases
LRS
Additive Effect
Significant LRS = 17.36
Suggestive LRS = 10.55
Sequence Site
Trait ID: HZI Thelp M430v2 (Feb11) RMA ** : 1457390_at
Mapping for Dataset: BXD, mapping on All Chromosomes
Using Haldane mapping function with no control for other QTLs
B
LRS
Megabases

Figure S4 Genome wide eQTL mapping of Prpf3 transcript in Treg and Th cells.

(A) eQTL map for *Prpf3* (probeset 1457390_at in Tregs and (B) in Th, respectively. See figure S3 for labels.

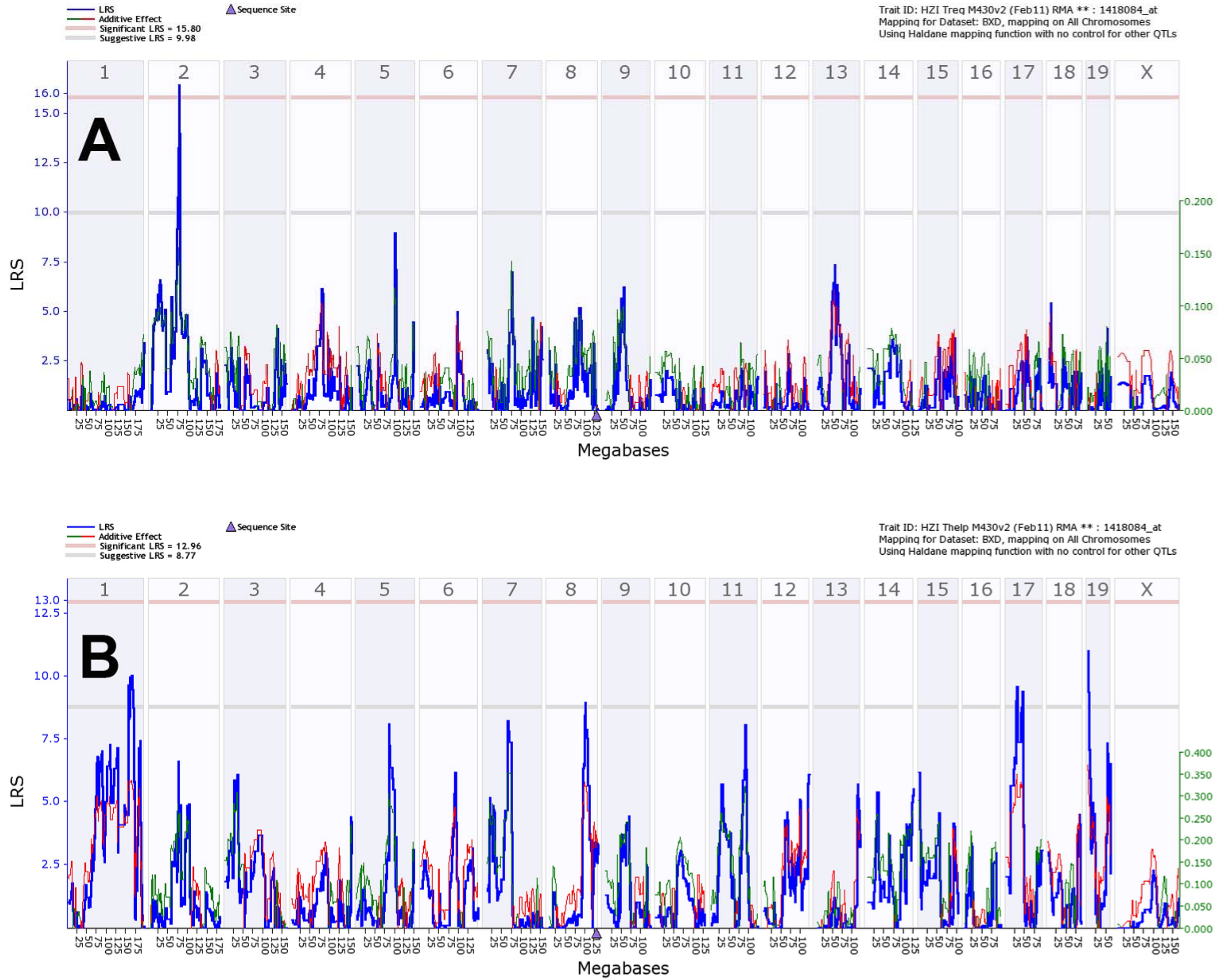
LRS
Additive Effect
Significant LRS = 15.80
Suggestive LRS = 9.98
Sequence Site
Trait ID: HZI Treg M430v2 (Feb11) RMA ** : 1418084_at
Mapping for Dataset: BXD, mapping on All Chromosomes
Using Haldane mapping function with no control for other QTLs
A
LRS
Megabases
1 2 3 4 5 6 7 8 9 10 11 12 13 14 15 16 17 18 19 X
LRS
Additive Effect
Significant LRS = 12.96
Suggestive LRS = 8.77
Sequence Site
Trait ID: HZI Thelp M430v2 (Feb11) RMA ** : 1418084_at
Mapping for Dataset: BXD, mapping on All Chromosomes
Using Haldane mapping function with no control for other QTLs
B
LRS
Megabases
1 2 3 4 5 6 7 8 9 10 11 12 13 14 15 16 17 18 19 X

Figure S5 Genome wide eQTL mapping of Nrp1 transcript in Treg and Th cells.

(A) eQTL map for (A) *Nrp1* (1418084_at) in Tregs and (B) in Th, respectively. See figure S3 for labels.

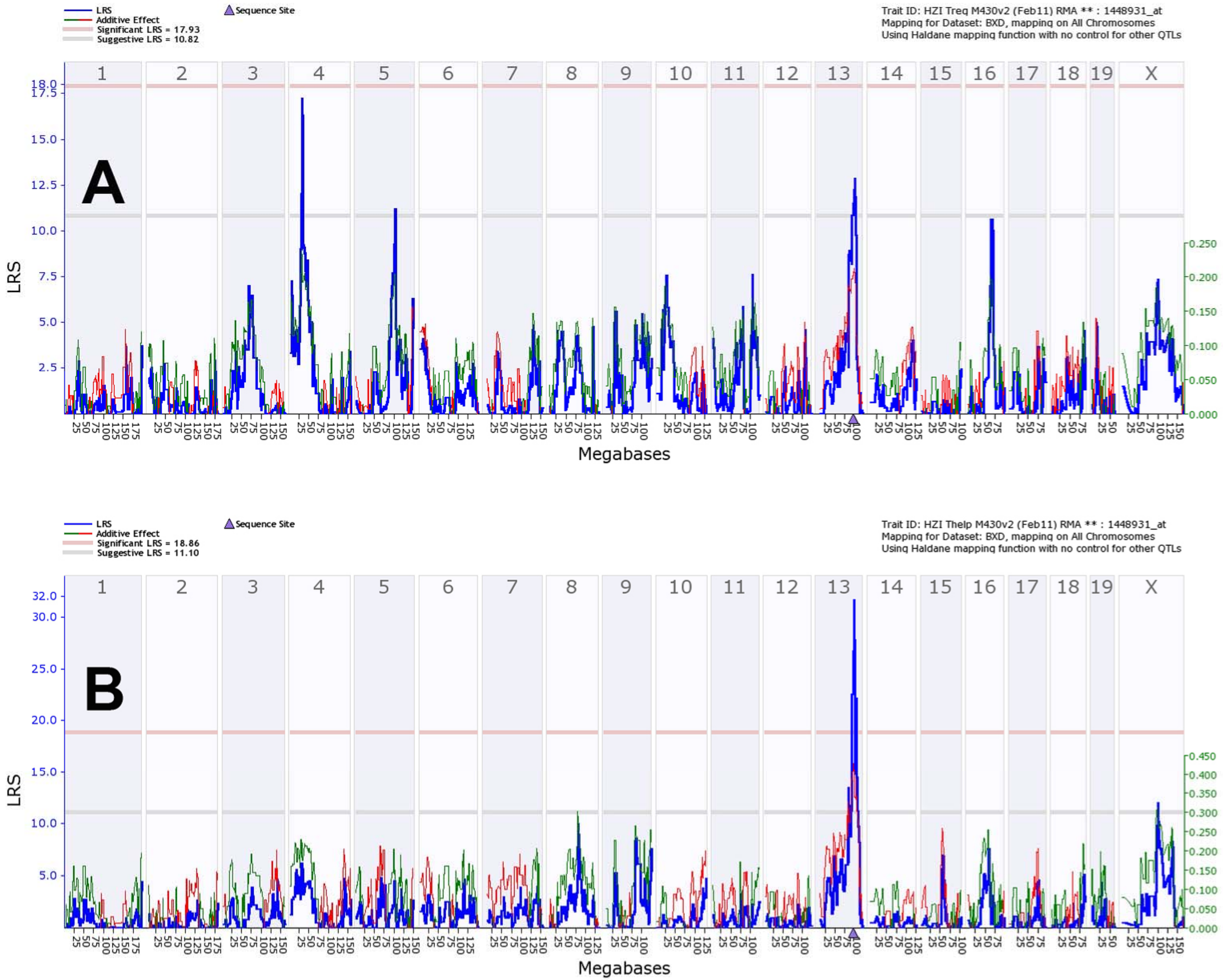

A
LRS
Additive Effect
Significant LRS = 17.93
Suggestive LRS = 10.82
Sequence Site
Trait ID: HZI Treg M430v2 (Feb11) RMA ** : 1448931_at
Mapping for Dataset: BXD, mapping on All Chromosomes
Using Haldane mapping function with no control for other QTLs
1 2 3 4 5 6 7 8 9 10 11 12 13 14 15 16 17 18 19 X
LRS
Megabases
B
LRS
Additive Effect
Significant LRS = 18.86
Suggestive LRS = 11.10
Sequence Site
Trait ID: HZI Thelp M430v2 (Feb11) RMA ** : 1448931_at
Mapping for Dataset: BXD, mapping on All Chromosomes
Using Haldane mapping function with no control for other QTLs
1 2 3 4 5 6 7 8 9 10 11 12 13 14 15 16 17 18 19 X
LRS
Megabases

Figure S6 Genome wide eQTL mapping of F2rl1 transcript in Treg and Th cells.

(A) eQTL map for ***F2rl1*** (1448931_at) in Tregs and (B) in Th, respectively. See figure S3 for labels.

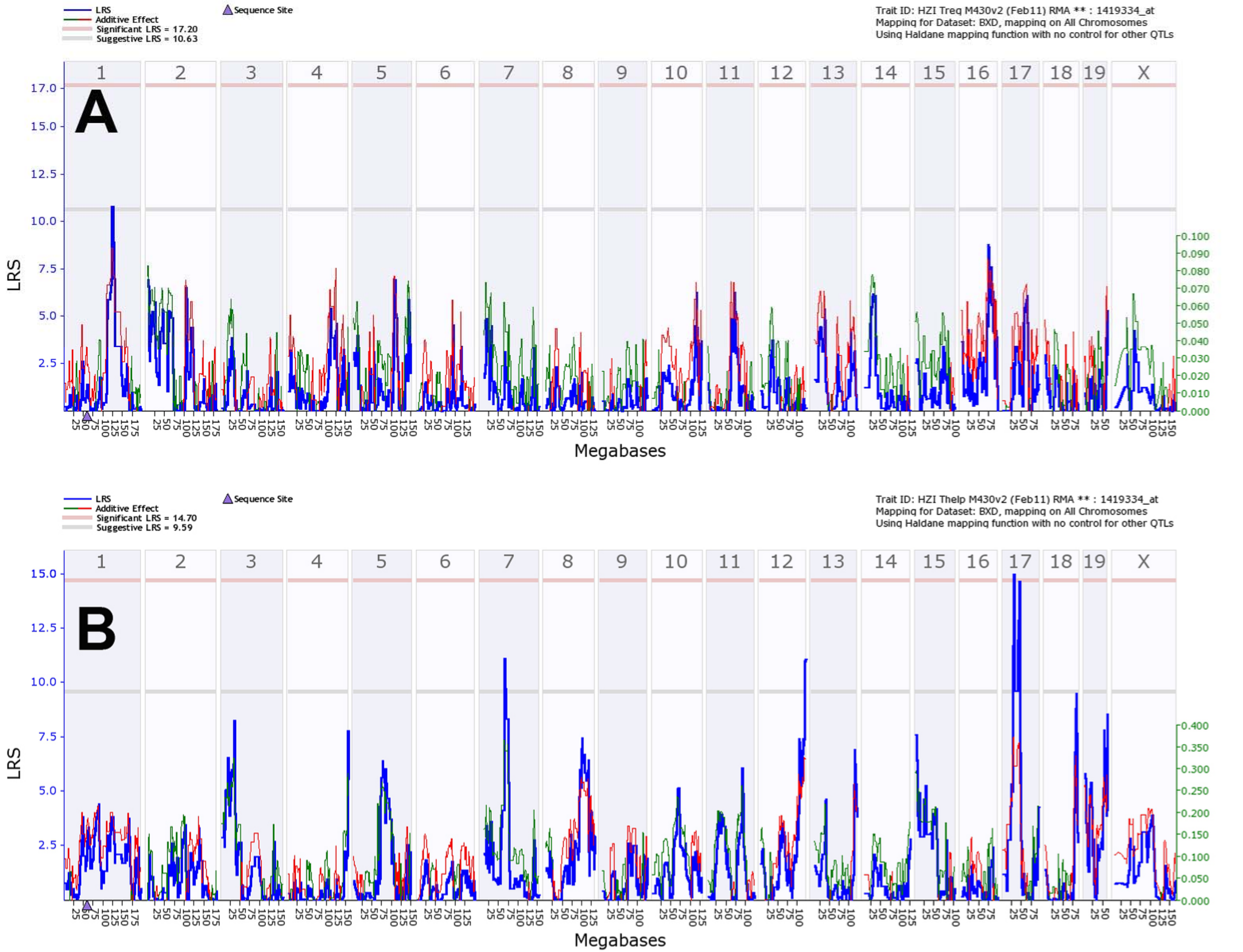

LRS
Additive Effect
Significant LRS = 17.20
Suggestive LRS = 10.63
Sequence Site
Trait ID: HZI Treg M430v2 (Feb11) RMA ** : 1419334_at
Mapping for Dataset: BXD, mapping on All Chromosomes
Using Haldane mapping function with no control for other QTLs
A
LRS
Megabases
LRS
Additive Effect
Significant LRS = 14.70
Suggestive LRS = 9.59
Sequence Site
Trait ID: HZI Thelp M430v2 (Feb11) RMA ** : 1419334_at
Mapping for Dataset: BXD, mapping on All Chromosomes
Using Haldane mapping function with no control for other QTLs
B
LRS
Megabases

Figure S7 Genome wide eQTL mapping of Ctla4 transcript in Treg and Th cells.

(A) eQTL map for *Ctla4* (probeset 1419334_at) in Tregs and (B) in Th, respectively. See figure S3 for labels.
