# Supplementary material for: Expression QTL mapping in regulatory and helper T cells from the BXD family of strains reveals novel cell-specific genes, gene-gene interactions and candidate genes for auto-immune disease
Source: BMC Genomics. 2011 Dec 19;12:610. doi: 10.1186/1471-2164-12-610 (PMC3277499; doi:10.1186/1471-2164-12-610)
Supplement: Additional file 5 — 'Additional Results and Discussion': This file contains additional results on: Trans-eQTLs mapping in Treg and Th cells, the analysis of candidate genes in trans-eQTL intervals, and the analysis of autoimmune traits. Also, the file contains additional discussion points on Treg-specifc genes, the analysis of the interval on chromosome 2 found to regulate expression of Nrp1, analysis of an interval on chromosome × regulating expression of Klrb1f/A630024B12Rik (killer cell lectin-like receptor subfamily B member 1F) and Ctla4 (cytotoxic T-lymphocyte-associated protein 4), the analysis of known Treg functional genes for possible cis- or trans-eQTLs, and the analysis of the QTLs interval chromosome 4 associated with autoimmune traits. [file 1471-2164-12-610-S5.PDF]

Additional Results and Discussion

Results

Trans-eQTL mapping reveals different mechanisms of gene regulation in Treg and Th cells

For the 30 top probesets that exhibited a trans-eQTL with an LRS of larger than 18 in Tregs, we found four probesets that also had an LRS of >=6 in Th cells at the same positions. Thus 26 out of the top Treg trans-eQTLs can be considered Treg-specific.

Analysis of candidate genes in trans-eQTL intervals reveals novel gene-gene regulatory interactions

Klrb1f / A630024B12Rik (killer cell lectin-like receptor subfamily B member 1F) was found to be expressed in both Treg and Th cells, but regulated in trans only in Treg cells by an interval on chromosome X (data not shown). *Klrb1f* was reported play a role in APC-mediated T cell co-stimulation, maintenance and memory [1]. The eQTL region for *Klrb1f* (90 – 110 Mb) contains 101 probesets which were well expressed (> 8) in Treg cells. Of these, *Chic1* (cysteine-rich hydrophobic domain 1) showed strong cis-eQTLs (LRS >= 15) and thus are the most likely candidates for regulating the expression of *Klrb1f* in Treg cells.

An interval on chromosome 2 (75 – 80 Mb) was found to regulate expression of *Nrp1*. This region contains 18 probesets which were also well expressed (> 8) in Treg cells (additional Table S13, see below). Of these, *Drbp1/Rbm45* (RNA binding motif protein 45) and *Agps* (alkylglycerone phosphate synthase) represented strong cis-eQTLs (LRS >= 15) and thus are the most likely candidates for regulating the expression of *Nrp1* in Treg cells.

Furthermore, *Ctla4* was expressed in both Treg and Th cells but significantly higher in Treg cells. In addition, it exhibited a significant trans-eQTL in Th on chromosome 17, but not in Treg cells. This region on chromosome 17 (25 – 40 Mb) contains 438 probesets which were also well expressed and exhibited a cis-eQTL with an LRS of >= 15.

Analysis of autoimmune traits

The QTL interval (105 – 130 Mb) on chromosome 4 which was associated with elevated rheumatoid factor levels contains 308 probesets expressed in Treg cells and 310 probesets were expressed in Th cells (> 8), respectively. 32 probesets were identified that also exhibited a

strong cis-eQTLs (LRS >= 15) in Treg cells (additional Table S14, see below). The peak interval (102 – 120 Mb) contained the genes *Tmem48 / 2810475A17Rik* (transmembrane protein 48), *Atpaf1* (ATP synthase mitochondrial F1 complex assembly factor 1), *Ptprf* (protein tyrosine phosphatase, receptor type, F), *Cdc20* (cell division cycle 20 homolog (*S. cerevisiae*)), *Gpbp1l1 / 5330440M15Rik* (GC-rich promoter binding protein 1-like 1), *Slc5a9* (solute carrier family 5 (sodium/glucose cotransporter), member 9), *Med8* (mediator of RNA polymerase II transcription, subunit 8 homolog (yeast)), and *Foxj3* (forkhead box J3). The cis-eQTL signals for *Slc5a9* and *Foxj3* were most likely caused by SNPs in the hybridization probe. Thus these genes, with the exception of *Slc5a9 and Foxj3* represent the most likely candidates for regulating this trait in Treg cells.

Discussion

The genes *Foxp3*, *Nrp1*, *Clta4*, *Tnfrsf18* / *Gitr*, *Il2ra* / *Cd25*, *Itgae* / *CD103*, *Ikzf4* / *Eos*, *Gpr83* have been reported to be hallmark genes for Treg cells [2, 3]. Indeed, all of them appear as DE-2fold genes specifically higher expressed in Treg cells.

In addition to *Nrp1*, *Gpr83* has been identified by comparative transcriptome analysis to be overexpressed in thymus-derived Tregs [3, 4]. Whereas transduction of naive Th cells with *Gpr83*-encoding retroviruses did not confer in vitro suppressive activity, we could show that *Gpr83*-transduced T cells were able to inhibit *in vivo* the effector phase of a severe contact hypersensitivity reaction of the skin. Moreover, this *in vivo* acquisition of suppressive activity was associated with the induction of *Foxp3* expression in *Gpr83*-transduced $CD4^+$ T cells under inflammatory conditions, thereby suggesting that *Gpr83* might be involved in the peripheral generation of *Foxp3*$^+$ Treg cells *in vivo* [3]. However, data obtained in other experimental systems revealed that *Gpr83* is dispensable for Treg development and function [5], thus the exact role of this molecule remains to be elucidated in more detail. This issue is further complicated by the existence of different splice variants. Of note, *Gpr83* isoform-4, which is predominantly expressed in Tregs, but not *Gpr83* isoform-1 retrovirally transduced T cells were able to interfere with inflammatory responses in vivo. Moreover, in vivo acquisition of suppressive activity is associated with the induction of Treg-associated molecules including Foxp3 in *Gpr83* isoform-4 but not *Gpr83* isoform-1 transduced $CD4^+$ T cells under inflammatory conditions [6].

Eos/Ikzf4 (Ikaros family zinc-finger transcription factor 4) has been shown to directly interact with *Foxp3* and recruit *Ctbp1* (C-terminal binding protein 1) to the protein complex (Pan et al. 2009). These genes are strongly expressed in Treg cells and with significantly higher levels in Treg compared to Th cells. *Foxp3* and *Eos* were expressed at 43- and 6-fold higher levels in Treg compared to Th cells, respectively. *Ctbp1* and *Nfat5* were only slightly (1.3 and 1.2-fold, respectively) higher expressed in Treg cells. *Eos* was demonstrated to serve as a co-repressor to recruit *Ctbp1* associated repression complexes to the promoter region of *Foxp3* target genes resulting in gene silencing. *Eos* knockdown reversed the transcriptional repression of genes that are targeted by Foxp3 suggesting that Eos plays an essential and selective role in Treg function through the recruitment of *Ctbp1* and its associated negative transcriptional regulators, thereby allowing selective gene silencing in Tregs [7]. The finding that abrogation of Treg activity is abrogated upon Eos knockdown demonstrates that gene silencing is critical to Treg function. This raises the possibility that the interaction between *Foxp3* with its co-repressors might be a potential therapeutic target for targeted restoration of anti-tumor or pathogen-specific immunity, for instance in the setting of cancer or chronic infections.
It should be noted that the Treg and Th cell populations were isolated based on two cell surface markers, CD4 and CD25. Thus, both cell populations may still be heterogeneous, especially the Th cell population.

An interval on chromosome 2 was found to regulate expression of *Nrp1*. In the murine system, *Nrp1* is specifically expressed in Treg cells, but completely absent in naïve and activated Th cells [8] which is well in line with the presence of a significant trans-eQTL Nrp1 in Tregs but not in Th cells. Whereas in mice *Nrp1* represents a highly specific and activation independent surface marker for Tregs, expression of this gene is not specific for human Treg cells. However, it might represent a novel activation marker of human T cells both *in vitro* and *in vivo* [9]. It acts a receptor for TGF-beta1, activates its latent form, and is relevant to regulatory T cell activity [10]. *Nrp1* is transiently expressed in thymus during T cell differentiation and is synchronized with CD25 down-regulation [11, 12]. *Nrp1* mutant mice display increased sensitivity to experimental autoimmune encephalomielitis [13]. *Nrp1* is essential for proper maintenance of peripheral tolerance and its absence can result in autoreactive responses [14]. Several candidate genes in the QTL interval on chromosome 2 exhibited a cis-eQTL and thus represent potential regulators. However, the biological function of these genes is not known or has thus far not been associated with the regulation immune responses.

Klrb1f / A630024B12Rik (killer cell lectin-like receptor subfamily B member 1F) was expressed in both Treg and Th cells, but regulated only in Treg cells by an interval on chromosome X. Two cis-eQTL regulated genes in this interval may thus be involved in the regulation of *Klrb1f*. The expression of the *Arr3* (arrestin 3, retinal) gene in macrophages is thought to modify innate immune responses via G-protein coupled receptor kinases [15]. *Arr3* is also involved in the endocytosis of the Ccr7 receptor [16]. The *Chic1 / Brx* (cysteine-rich hydrophobic domain 1) gene is essential for the expression of *Nfat5*, another important immune response gene [17].
Furthermore, *Ctla4* was expressed in both Treg and Th cells but significantly higher in Treg cells. In addition, it exhibited a significant trans-eQTL in Th but not in Treg cells. Whereas Tregs show constitutively high *Ctla4* expression, expression of this molecule is induced in activated Th cells, [18], a process critically involved in calming down of effector T cell responses after the elimination of pathogenic invaders. Presence of a significant trans-eQTL exclusively in Th but not Treg cells suggests different circuits regulating expression of this particular gene in both cellular subsets. *Ctla4* has been proven to contribute to Treg function [19] and compelling evidence exist for the pathogenetical involvement of CTLA4 in different autoimmune diseases and several studies suggest disease-associated polymorphisms [20].

Also, we investigated all known Treg functional genes for possible cis- or trans-eQTLs. No eQTL was found for the most important Treg gene, *Foxp3*. The absence of a cis-eQTL suggests that there is no functional polymorphism in the gene itself that influences the expression levels between the DBA/2J and C57BL/6J alleles. Indeed, no SNP has been identified in the transcribed region of the *Foxp3* gene between DBA/2J and C57BL/6J. Therefore, it is very unlikely to identify direct Foxp3 downstream effects as eQTLs in the transcriptome of BXD mice. Also, no significant trans-eQTL was found that may regulate *Foxp3* gene expression. The most likely reason is that there are no polymorphisms between DBA/2J and C57BL/6J which affect the function of Foxp3 upstream regulatory genes.

Two QTLs intervals were previously identified on chromosome 2 and 4 that were associated with autoimmune traits [21]. The QTL interval on chromosome 4 regulating contained several cis-eQTL genes in Treg cells: *Tmem48, Atpaf1, Cdc20, Gpbp1l1,* and *Med8*. *Med8* (mediator of RNA polymerase II transcription, subunit 8 homolog (yeast)). It has been shown that inhibition of host transcription and the interferon response by viral proteins is linked to interaction with *Med8*

[22] suggesting an immune-modulating function of this gene. *Tmem48* (transmembrane protein 48, also *NDC1* in human) is involved in the localization of the nuclear pore complexes [23]. The *Cdc20* (cell division cycle 20 homolog (*S. cerevisiae*)) gene represents a key regulator of the cell cycle, differentiation and apoptosis [24]. But no immune function has yet been described for these genes. No biological functions have been yet been described for *Gpbp1l1 / 5330440M15Rik* (GC-rich promoter binding protein 1-like 1) and *Atpaf1* (ATP synthase mitochondrial F1 complex assembly factor 1). *Ptprf* (protein tyrosine phosphatase, receptor type, F) is a regulator of insulin signaling and also promotes neurite outgrowth. Furthermore, *Ptprf* is an important modulator of TCR signaling that controls thymocyte differentiation [25, 26]. Deficiency of *Ptprf* affected the differentiation of immature thymocytes [27].

References

1. Tian W, Nunez R, Cheng S, Ding Y, Tumang J, Lyddane C, Roman C, Liou HC: **C-type lectin OCILRP2/Clr-g and its ligand NKRP1f costimulate T cell proliferation and IL-2 production**. *Cell Immunol* 2005, **234**(1):39-53.
2. Takahashi T, Tagami T, Yamazaki S, Uede T, Shimizu J, Sakaguchi N, Mak TW, Sakaguchi S: **Immunologic self-tolerance maintained by CD25(+)CD4(+) regulatory T cells constitutively expressing cytotoxic T lymphocyte-associated antigen 4**. *J Exp Med* 2000, **192**(2):303-310.
3. Hansen W, Loser K, Westendorf AM, Bruder D, Pfoertner S, Siewert C, Huehn J, Beissert S, Buer J: **G protein-coupled receptor 83 overexpression in naive CD4+CD25- T cells leads to the induction of Foxp3+ regulatory T cells in vivo**. *J Immunol* 2006, **177**(1):209-215.
4. Sugimoto N, Oida T, Hirota K, Nakamura K, Nomura T, Uchiyama T, Sakaguchi S: **Foxp3-dependent and -independent molecules specific for CD25+CD4+ natural regulatory T cells revealed by DNA microarray analysis**. *Int Immunol* 2006, **18**(8):1197-1209.
5. Lu LF, Gavin MA, Rasmussen JP, Rudensky AY: **G protein-coupled receptor 83 is dispensable for the development and function of regulatory T cells**. *Mol Cell Biol* 2007, **27**(23):8065-8072.
6. Hansen W, Westendorf AM, Toepfer T, Mauel S, Geffers R, Gruber AD, Buer J: **Inflammation in vivo is modulated by GPR83 isoform-4 but not GPR83 isoform-1 expression in regulatory T cells**. *Genes Immun* 2010, **11**(4):357-361.
7. Pan F, Yu H, Dang EV, Barbi J, Pan X, Grosso JF, Jinasena D, Sharma SM, McCadden EM, Getnet D *et al*: **Eos mediates Foxp3-dependent gene silencing in CD4+ regulatory T cells**. *Science* 2009, **325**(5944):1142-1146.
8. Bruder D, Probst-Kepper M, Westendorf AM, Geffers R, Beissert S, Loser K, von Boehmer H, Buer J, Hansen W: **Neuropilin-1: a surface marker of regulatory T cells**. *Eur J Immunol* 2004, **34**(3):623-630.
9. Milpied P, Renand A, Bruneau J, Mendes-da-Cruz DA, Jacquelin S, Asnafi V, Rubio MT, MacIntyre E, Lepelletier Y, Hermine O: **Neuropilin-1 is not a marker of human Foxp3+ Treg**. *Eur J Immunol* 2009, **39**(6):1466-1471.

10. Glinka Y, Prud'homme GJ: **Neuropilin-1 is a receptor for transforming growth factor beta-1, activates its latent form, and promotes regulatory T cell activity**. *J Leukoc Biol* 2008, **84**(1):302-310.
11. Lepelletier Y, Smaniotto S, Hadj-Slimane R, Villa-Verde DM, Nogueira AC, Dardenne M, Hermine O, Savino W: **Control of human thymocyte migration by Neuropilin-1/Semaphorin-3A-mediated interactions**. *Proc Natl Acad Sci U S A* 2007, **104**(13):5545-5550.
12. Corbel C, Lemarchandel V, Thomas-Vaslin V, Pelus AS, Agboton C, Romeo PH: **Neuropilin 1 and CD25 co-regulation during early murine thymic differentiation**. *Dev Comp Immunol* 2007, **31**(11):1082-1094.
13. Tian L, Rauvala H, Gahmberg CG: **Neuronal regulation of immune responses in the central nervous system**. *Trends Immunol* 2009, **30**(2):91-99.
14. Solomon BD, Mueller C, Chae WJ, Alabanza LM, Bynoe MS: **Neuropilin-1 attenuates autoreactivity in experimental autoimmune encephalomyelitis**. *Proc Natl Acad Sci U S A* 2011, **108**(5):2040-2045.
15. Loniewski K, Shi Y, Pestka J, Parameswaran N: **Toll-like receptors differentially regulate GPCR kinases and arrestins in primary macrophages**. *Mol Immunol* 2008, **45**(8):2312-2322.
16. Byers MA, Calloway PA, Shannon L, Cunningham HD, Smith S, Li F, Fassold BC, Vines CM: **Arrestin 3 mediates endocytosis of CCR7 following ligation of CCL19 but not CCL21**. *J Immunol* 2008, **181**(7):4723-4732.
17. Kino T, Takatori H, Manoli I, Wang Y, Tiulpakov A, Blackman MR, Su YA, Chrousos GP, DeCherney AH, Segars JH: **Brx mediates the response of lymphocytes to osmotic stress through the activation of NFAT5**. *Sci Signal* 2009, **2**(57):ra5.
18. Perkins D, Wang Z, Donovan C, He H, Mark D, Guan G, Wang Y, Walunas T, Bluestone J, Listman J *et al*: **Regulation of CTLA-4 expression during T cell activation**. *J Immunol* 1996, **156**(11):4154-4159.
19. Bour-Jordan H, Bluestone JA: **Regulating the regulators: costimulatory signals control the homeostasis and function of regulatory T cells**. *Immunol Rev* 2009, **229**(1):41-66.
20. Kristiansen OP, Larsen ZM, Pociot F: **CTLA-4 in autoimmune diseases--a general susceptibility gene to autoimmunity?** *Genes Immun* 2000, **1**(3):170-184.
21. Mountz JD, Yang P, Wu Q, Zhou J, Tousson A, Fitzgerald A, Allen J, Wang X, Cartner S, Grizzle WE *et al*: **Genetic segregation of spontaneous erosive arthritis and generalized autoimmune disease in the BXD2 recombinant inbred strain of mice**. *Scand J Immunol* 2005, **61**(2):128-138.
22. Leonard VH, Kohl A, Hart TJ, Elliott RM: **Interaction of Bunyamwera Orthobunyavirus NSs protein with mediator protein MED8: a mechanism for inhibiting the interferon response**. *J Virol* 2006, **80**(19):9667-9675.
23. Yamazumi Y, Kamiya A, Nishida A, Nishihara A, Iemura S, Natsume T, Akiyama T: **The transmembrane nucleoporin NDC1 is required for targeting of ALADIN to nuclear pore complexes**. *Biochem Biophys Res Commun* 2009, **389**(1):100-104.
24. Lin M, Chang JK, Shankar D, Sakamoto KM: **The role of p55CDC in cell cycle control and mammalian cell proliferation, differentiation, and apoptosis**. *Exp Mol Pathol* 2003, **74**(2):123-128.
25. Mander A, Hodgkinson CP, Sale GJ: **Knock-down of LAR protein tyrosine phosphatase induces insulin resistance**. *FEBS Lett* 2005, **579**(14):3024-3028.
26. Yang T, Bernabeu R, Xie Y, Zhang JS, Massa SM, Rempel HC, Longo FM: **Leukocyte antigen-related protein tyrosine phosphatase receptor: a small ectodomain isoform functions as a homophilic ligand and promotes neurite outgrowth**. *J Neurosci* 2003, **23**(8):3353-3363.

27. Kondo S, Kishi H, Muraguchi A: **Regulatory role of leukocyte-common-antigen-related molecule (LAR) in thymocyte differentiation**. *Eur J Immunol* 2010, **40**(5):1296-1302.
28. Fontenot JD, Gavin MA, Rudensky AY: **Foxp3 programs the development and function of CD4+CD25+ regulatory T cells**. *Nat Immunol* 2003, **4**(4):330-336.
29. Sakaguchi S, Sakaguchi N, Asano M, Itoh M, Toda M: **Immunologic self-tolerance maintained by activated T cells expressing IL-2 receptor alpha-chains (CD25). Breakdown of a single mechanism of self-tolerance causes various autoimmune diseases**. *J Immunol* 1995, **155**(3):1151-1164.
30. Shimizu J, Yamazaki S, Takahashi T, Ishida Y, Sakaguchi S: **Stimulation of CD25(+)CD4(+) regulatory T cells through GITR breaks immunological self-tolerance**. *Nat Immunol* 2002, **3**(2):135-142.
31. Collison LW, Workman CJ, Kuo TT, Boyd K, Wang Y, Vignali KM, Cross R, Sehy D, Blumberg RS, Vignali DA: **The inhibitory cytokine IL-35 contributes to regulatory T-cell function**. *Nature* 2007, **450**(7169):566-569.
32. Wing K, Onishi Y, Prieto-Martin P, Yamaguchi T, Miyara M, Fehervari Z, Nomura T, Sakaguchi S: **CTLA-4 control over Foxp3+ regulatory T cell function**. *Science* 2008, **322**(5899):271-275.
33. Liu G, Burns S, Huang G, Boyd K, Proia RL, Flavell RA, Chi H: **The receptor S1P1 overrides regulatory T cell-mediated immune suppression through Akt-mTOR**. *Nat Immunol* 2009, **10**(7):769-777.
34. Cao X, Cai SF, Fehniger TA, Song J, Collins LI, Piwnica-Worms DR, Ley TJ: **Granzyme B and perforin are important for regulatory T cell-mediated suppression of tumor clearance**. *Immunity* 2007, **27**(4):635-646.
35. Garin MI, Chu CC, Golshayan D, Cernuda-Morollon E, Wait R, Lechler RI: **Galectin-1: a key effector of regulation mediated by CD4+CD25+ T cells**. *Blood* 2007, **109**(5):2058-2065.
36. Lehmann J, Huehn J, de la Rosa M, Maszyna F, Kretschmer U, Krenn V, Brunner M, Scheffold A, Hamann A: **Expression of the integrin alpha Ebeta 7 identifies unique subsets of CD25+ as well as CD25- regulatory T cells**. *Proc Natl Acad Sci U S A* 2002, **99**(20):13031-13036.
37. Huang CT, Workman CJ, Flies D, Pan X, Marson AL, Zhou G, Hipkiss EL, Ravi S, Kowalski J, Levitsky HI *et al*: **Role of LAG-3 in regulatory T cells**. *Immunity* 2004, **21**(4):503-513.
38. Borsellino G, Kleinewietfeld M, Di Mitri D, Sternjak A, Diamantini A, Giometto R, Hopner S, Centonze D, Bernardi G, Dell'Acqua ML *et al*: **Expression of ectonucleotidase CD39 by Foxp3+ Treg cells: hydrolysis of extracellular ATP and immune suppression**. *Blood* 2007, **110**(4):1225-1232.
39. Deaglio S, Dwyer KM, Gao W, Friedman D, Usheva A, Erat A, Chen JF, Enjyoji K, Linden J, Oukka M *et al*: **Adenosine generation catalyzed by CD39 and CD73 expressed on regulatory T cells mediates immune suppression**. *J Exp Med* 2007, **204**(6):1257-1265.
40. Chaudhry A, Rudra D, Treuting P, Samstein RM, Liang Y, Kas A, Rudensky AY: **CD4+ regulatory T cells control TH17 responses in a Stat3-dependent manner**. *Science* 2009, **326**(5955):986-991.
41. Zheng Y, Chaudhry A, Kas A, deRoos P, Kim JM, Chu TT, Corcoran L, Treuting P, Klein U, Rudensky AY: **Regulatory T-cell suppressor program co-opts transcription factor IRF4 to control T(H)2 responses**. *Nature* 2009, **458**(7236):351-356.
42. Cretney E, Xin A, Shi W, Minnich M, Masson F, Miasari M, Belz GT, Smyth GK, Busslinger M, Nutt SL *et al*: **The transcription factors Blimp-1 and IRF4 jointly control the differentiation and function of effector regulatory T cells**. *Nat Immunol* 2011, **12**(4):304-311.
43. Chen X, Subleski JJ, Kopf H, Howard OM, Mannel DN, Oppenheim JJ: **Cutting edge: expression of TNFR2 defines a maximally suppressive subset of mouse CD4+CD25+FoxP3+ T regulatory cells: applicability to tumor-infiltrating T regulatory cells**. *J Immunol* 2008, **180**(10):6467-6471.

44. Sugai M, Aoki K, Osato M, Nambu Y, Ito K, Taketo MM, Shimizu A: **Runx3 is required for full activation of regulatory T cells to prevent colitis-associated tumor formation**. *J Immunol* 2011, **186**(11):6515-6520.
45. Kuczma M, Lee JR, Kraj P: **Connexin 43 signaling enhances the generation of foxp3+ regulatory T cells**. *J Immunol* 2011, **187**(1):248-257.
46. Patterson SJ, Han JM, Garcia R, Assi K, Gao T, O'Neill A, Newton AC, Levings MK: **Cutting Edge: PHLPP Regulates the Development, Function, and Molecular Signaling Pathways of Regulatory T Cells**. *J Immunol* 2011, **186**(10):5533-5537.
47. Probst-Kepper M, Geffers R, Kroger A, Viegas N, Erck C, Hecht HJ, Lunsdorf H, Roubin R, Moharregh-Khiabani D, Wagner K *et al*: **GARP: a key receptor controlling FOXP3 in human regulatory T cells**. *J Cell Mol Med* 2009, **13**(9B):3343-3357.
48. Wang R, Kozhaya L, Mercer F, Khaitan A, Fujii H, Unutmaz D: **Expression of GARP selectively identifies activated human FOXP3+ regulatory T cells**. *Proc Natl Acad Sci U S A* 2009, **106**(32):13439-13444.
49. Tran DQ, Andersson J, Hardwick D, Bebris L, Illei GG, Shevach EM: **Selective expression of latency-associated peptide (LAP) and IL-1 receptor type I/II (CD121a/CD121b) on activated human FOXP3+ regulatory T cells allows for their purification from expansion cultures**. *Blood* 2009, **113**(21):5125-5133.
50. Kubach J, Lutter P, Bopp T, Stoll S, Becker C, Huter E, Richter C, Weingarten P, Warger T, Knop J *et al*: **Human CD4+CD25+ regulatory T cells: proteome analysis identifies galectin-10 as a novel marker essential for their anergy and suppressive function**. *Blood* 2007, **110**(5):1550-1558.
51. Tone Y, Furuuchi K, Kojima Y, Tykocinski ML, Greene MI, Tone M: **Smad3 and NFAT cooperate to induce Foxp3 expression through its enhancer**. *Nat Immunol* 2008, **9**(2):194-202.

Tables

Table S13 Probesets of genes located in the QTL interval on chromosome 2 regulating the expression of Nrp1 and exhibiting an expression signal larger than 8.

Probeset	Symbol	Description	Location (Chr, Mb)	Mean Expr	Max LRS	Max LRS Location (Chr: Mb)
1428262_s_at	*Hnrpa3*	heterogeneous nuclear ribonucleoprotein A3; last intron or distal 3' UTR	Chr2: 75.503875	13.71	12.5	Chr12: 16.835275
1452712_at	*Hnrpa3*	heterogeneous nuclear ribonucleoprotein A3	Chr2: 75.504797	11.79	10.7	Chr1: 38.079087
1452774_at	*Hnrpa3*	heterogeneous nuclear ribonucleoprotein A3; distal half of 3' UTR	Chr2: 75.506903	13.31	17.4	Chr3: 54.674449
1416543_at	*Nfe2l2*	nuclear factor, erythroid derived 2, like 2 (lung damage associated); last exon and 3' UTR	Chr2: 75.513676	9.89	14.7	Chr1: 122.017244
1454918_at	*Agps*	alkylglycerone phosphate synthase; distal 3'UTR	Chr2: 75.767164	9.82	31.7	Chr2: 75.856729
1434919_at	*Agps*	alkylglycerone phosphate synthase	Chr2: 75.768807	9.35	19.9	Chr2: 75.856729
1437904_at	*Drbp1*	developmentally regulated RNA binding protein 1; last two exons including mid and distal 3' UTR	Chr2: 76.218483	10.27	96.6	Chr2: 75.856729
1448923_at	*Prkra*	protein kinase, interferon inducible double stranded RNA dependent activator	Chr2: 76.468177	8.57	22	Chr1: 157.588921

1420840_at	*Plekha3*	pleckstrin homology domain-containing, family A (phosphoinositide binding specific) member 3; distal half of 3' UTR	Chr2: 76.534071	9.70	13	Chr6: 95.210718
1427445_a_at	*Ttn*	titin	Chr2: 76.542185	10.45	9.9	Chr16: 31.615881
1427446_s_at	*Ttn*	titin	Chr2: 76.542262	8.19	15.4	Chr3: 37.307265
1455549_at	*Sestd1*	SEC14 and spectrin domains 1; far 3' UTR (or possible small intercalated gene 3' of Sestd1)	Chr2: 77.018665	8.52	11.1	Chr11: 119.216123
1448670_at	*Ube2e3*	ubiquitin-conjugating enzyme E2E 3, UBC4/5 homolog; 3' UTR	Chr2: 78.760235	10.72	15.1	Chr7: 30.566470
1457376_at	*Itga4*	integrin alpha 4 (antigen CD49D, alpha 4 subunit of VLA-4 receptor); intron	Chr2: 79.159848	9.61	11.4	Chr2: 77.938377
1421194_at	*Itga4*	integrin alpha 4 (antigen CD49D, alpha 4 subunit of VLA-4 receptor); mid 3' UTR	Chr2: 79.168370	8.96	11.1	Chr3: 155.062196
1456498_at	*Itga4*	integrin alpha 4 (antigen CD49D, alpha 4 subunit of VLA-4 receptor); mid distal 3' UTR	Chr2: 79.170427	10.80	11.2	Chr10: 19.804439
1436037_at	*Itga4*	integrin, alpha 4 (antigen CD49D, alpha 4 subunit of VLA-4 receptor); distal 3' UTR	Chr2: 79.172683	12.19	7.9	Chr6: 136.698274
1423613_at	*Ssfa2*	sperm specific antigen 2; mid 3' UTR	Chr2: 79.512251	8.98	11.4	Chr15: 92.839468

Probeset: probeset ID of microarray hybridization probe, Symbol: gene symbol in GeneNetwork (note that this may differ from the gene description in MGI), Description: gene description in GeneNetwork (note that this may differ from the gene description in MGI), Location: chromosomal location Chromosome, Megabase of gene, Mean Expr: mean expression level in respective cell type as $\log_2$ value. Max LRS value of maximum LRS, Max LRS location: position of QTL (Chromosome, Megabase) exhibiting the maximum LRS value.

Table S14 Probesets of genes located in the QTL interval on chromosome 4 (elevated RF levels), expressed in Treg and exhibiting a cis-eQTL of LRS >=15.

Probeset	Symbol	Description	Location (Chr, Mb)	Mean Expr	Max LRS	Max LRS Location (Chr: Mb)
1433813_at	*2810475A17Rik*	RIKEN cDNA 2810474O19 gene	Chr4: 107.088394	8.10	33.4	Chr4: 105.321483
1429879_at	*0610037L13Rik*	RIKEN cDNA 0610037L13 gene	Chr4: 107.569424	8.10	17.3	Chr4: 105.321483
1440779_s_at	*Slc5a9*	solute carrier family 5 (sodium/glucose cotransporter), member 9	Chr4: 111.549922	9.36	25.9	Chr4: 119.150595
1434934_at	*Atpaf1*	ATP synthase mitochondrial F1 complex assembly factor 1; mid 3' UTR	Chr4: 115.483950	9.98	38.1	Chr4: 108.879987
1436358_at	*Atpaf1*	ATP synthase mitochondrial F1 complex assembly factor 1; distal 3' UTR	Chr4: 115.486310	10.20	26.5	Chr4: 119.150595
1449113_at	*5330440M15Rik*	RIKEN cDNA 5330440M15 gene	Chr4: 116.265744	9.21	52.2	Chr4: 110.966847
1420842_at	*Ptprf*	protein tyrosine phosphatase, receptor type, F	Chr4: 117.880911	8.35	51.7	Chr4: 110.966847
1420843_at	*Ptprf*	protein tyrosine phosphatase, receptor type, F; 3' UTR	Chr4: 117.881617	8.31	46.3	Chr4: 108.879987
1442417_at	*Med8*	mediator of RNA polymerase II transcription, subunit 8 homolog (yeast)	Chr4: 118.087787	8.17	60	Chr4: 119.150595
1439377_x_at	*Cdc20*	cell division cycle 20; last exon and	Chr4: 118.105545	11.10	23.3	Chr4: 110.966847

		proximal 3' UTR				
1416664_at	*Cdc20*	cell division cycle 20; exons 7 through 10	Chr4: 118.105712	9.95	23.7	Chr4: 108.879987
1438220_at	*Foxj3*	forkhead box J3	Chr4: 119.299430	8.09	73.5	Chr4: 119.150595
1422467_at	*Ppt1*	palmitoyl-protein thioesterase 1 (neuronal ceroid lipofuscinosis); 3' UTR of Ppt1 and antisense in Cap1 far 3' UTR	Chr4: 122.535659	10.38	25.6	Chr4: 122.536808
1417461_at	*Cap1*	CAP, adenylate cyclase-associated protein 1; distal 3' UTR (test Mendelian 4.122)	Chr4: 122.536488	11.75	76	Chr4: 122.536808
1417462_at	*Cap1*	CAP, adenylate cyclase-associated protein 1; exons 9, 11, 13 and proximal 3' UTR (test Mendelian 4.122)	Chr4: 122.537308	8.17	63.8	Chr4: 122.536808
1430100_at	*Mrps15*	mitochondrial ribosomal protein S15	Chr4: 125.728896	9.41	19.2	Chr4: 125.265631
1452125_at	*Thrap3*	thyroid hormone receptor associated protein 3; intron 3	Chr4: 125.856036	12.18	30.2	Chr4: 125.265631
1416852_a_at	*Ncdn*	neurochondrin; distal 3' UTR	Chr4: 126.421015	9.92	16.4	Chr4: 129.249414
1438685_at	*Zmym6*	zinc finger, MYM-type 6; last exon	Chr4: 126.801020	9.51	19.3	Chr4: 125.265631
1439145_at	*Lck*	lymphocyte protein tyrosine kinase	Chr4: 129.234128	11.75	36.2	Chr4: 129.249414
1457917_at	*Lck*	lymphocyte protein tyrosine	Chr4: 129.248111	12.26	16.1	Chr4: 129.249414

		kinase				
1425054_a_at	*Tmem234*	transmembrane protein 234; last three exons and proximal 3' UTR	Chr4: 129.279445	11.49	27.5	Chr4: 127.074543
1452356_at	*Iqcc*	IQ motif containing C	Chr4: 129.293095	8.25	60.3	Chr4: 129.249414
1459840_s_at	*Ccdc28b*	coiled coil domain containing 28B; last exon; end of last exon and proximal 3'UTR	Chr4: 129.296559	8.87	20.3	Chr4: 129.249414
1434157_at	*Txln*	TXK tyrosine kinase	Chr4: 129.304606	8.92	28	Chr4: 129.249414
1434541_x_at	*Khdrbs1*	KH domain containing, RNA binding, signal transduction associated 1; distal 3' UTR	Chr4: 129.392134	12.85	24.4	Chr4: 129.249414
1438462_x_at	*Khdrbs1*	KH domain containing, RNA binding, signal transduction associated 1; distal 3' UTR	Chr4: 129.392134	8.82	19.6	Chr4: 125.265631
1440434_at	*6230424H07Rik*	ESTs, Weakly similar to RIKEN cDNA 5730493B19 [] [M.musculus]	Chr4: 129.510448	8.80	36.5	Chr4: 129.249414
1435129_at	*Ptp4a2*	protein tyrosine phosphatase 4a2; highly expressed message, antisense in 5' UTR and exon 1	Chr4: 129.516447	13.78	59.4	Chr4: 129.249414
1420612_s_at	*Ptp4a2*	protein tyrosine phosphatase 4a2	Chr4: 129.525763	8.29	20.9	Chr4: 127.074543
1460707_at	*Ptp4a2*	protein tyrosine phosphatase 4a2; distal 3'	Chr4: 129.526708	13.40	18.3	Chr4: 129.249414

		UTR				
1444188_at	*Ptp4a2*	protein tyrosine phosphatase 4a2	Chr4: 129.527854	8.14	64.1	Chr4: 129.249414

See Table S13 for descriptions of columns.
